# Global burden of ischemic heart disease attributable to dietary risks in young adults, 1990–2021: trends and future projections

**DOI:** 10.3389/fpubh.2026.1729653

**Published:** 2026-02-13

**Authors:** Yao Liang, Xiaoyu Zhang, Mengli Duan, Chenglong Hu, Hui Li, Min Yang

**Affiliations:** 1 The Second Department of Critical Care Medicine, The Second Affiliated Hospital of Anhui Medical University, Hefei, Anhui, China; 2 The Laboratory of Cardiopulmonary Resuscitation and Critical Care Medicine, The Second Affiliated Hospital of Anhui Medical University, Hefei, Anhui, China

**Keywords:** dietary risks, forecast, global burden of disease, ischemic heart disease, young adults

## Abstract

**Background:**

Ischemic heart disease (IHD) is imposing a growing global burden on young adults, for whom dietary factors are a prominent and feasible preventive target. This study investigated the global burden and trends of IHD attributable to dietary risks among young adults from 1990 to 2021, with projections to 2031.

**Methods:**

Data for this study, sourced from Global Burden of Disease (GBD) 2021, comprehensively analyzed the burden of ischemic heart disease (IHD) due to dietary factors in young adults. Temporal trends were evaluated using estimated annual percentage changes (EAPCs), while future trends were projected using an Auto-Regressive Integrated Moving Average (ARIMA) model.

**Results:**

Globally in 2021, the dietary-related IHD mortality and DALYs rates among young adults were 9.48 (95% UI: −1.54 to 13.41) and 465.57 (95% UI: −78.20 to 658.72) respectively, with males bearing a heavier burden than females. From 1990 to 2021, both mortality and DALYs rates demonstrated consistent declines. The low-middle SDI region and Eastern Europe exhibited the highest IHD burden among the five SDI regions and 21 GBD regions, respectively. Diet low in whole grains was identified as the leading dietary risk factor for IHD burden, whereas high consumption of sugar-sweetened beverages remained the only dietary risk factor demonstrating an upward trend. Projections suggest that by 2031, the global dietary-related IHD mortality rate among young adults will decrease by 4.58%, while the DALYs rate is expected to increase by 0.34%.

**Conclusion:**

This finding underscores the critical need for targeted dietary interventions aimed at reducing IHD risk, particularly among young adults on a global scale.

## 1 Introduction

Ischemic heart disease (IHD) refers to a cardiovascular disease characterized by a discrepancy between the supply of oxygen to the myocardium and its demand, which is caused by coronary atherosclerosis with luminal stenosis and endothelial dysfunction (1). In young adults, endothelial dysfunction not only impairs coronary vasodilation by reducing nitric oxide bioavailability and enhancing endothelin-1-mediated constriction, but also promotes thrombosis through increased expression of key coagulation factors and inhibition of the fibrinolytic system. Furthermore, these functional alterations can precipitate acute thrombus formation on minimally stenotic plaques and induce myocardial ischemia without critical fixed stenosis (2, 3). According to World Health Organization (WHO), IHD contributes to 13% of all deaths globally. Since 2000, the number of deaths from IHD has been continuously increasing, with an increase of 2.7 million to 9.1 million in 2021 (4). In 2019, among people aged 15–49, the mortality rate and DALYs rate were 15.88 and 777.07 per 100,000 individuals, respectively (5). The burden of IHD is increasingly trending towards younger age groups (5, 6).

Dietary risks—including high intake of fat, sugar, and salt—increase the risk of cardiovascular disease (CVD) by elevating low-density lipoprotein cholesterol (LDL-C), impairing vascular endothelial function, and enhancing oxidative stress (7). This risk is particularly pertinent given the prevalent preference among contemporary young people for sweet and salty foods, a dietary habit that significantly contributes to the rising incidence of IHD in this population (8). Relevant data indicated that in 2019, worldwide statistic of death rates from IHD attributed to dietary risks was 10.98 per 100,000 population aged 25 to 49, and the proportion affected by dietary risks is increasing (5).

However, there is currently a lack of research on the global distribution of IHD related to dietary risks specifically among young people. To fill this gap, this study aims to use data from the 2021 Global Study of Disease, Injury, and Risk Factors (GBD) to analyze the global burden of IHD among young adults due to 13 dietary risk factors, examine the differences between different demographics and socioeconomic groups among young people, and better understand the prevalence of ischemic heart disease related to dietary risk, ultimately guiding the formulation of dietary guidelines for the prevention and management of IHD among young people.

## 2 Methods

### 2.1 Data sources and definitions

GBD 2021 represents an integrated, collaborative surveillance system involving multiple nations, comprehensively quantifying health losses and providing exhaustive and systematic estimates of 371 diseases and injuries, as well as 88 risk factors from 1990 to 2021 (9, 10). Owing to the unavailability of dietary-attributable IHD estimates for the 15–24 age group in the GBD database, this study focused on adults aged 25–49 years, representing the core working-age population at their physiological prime, characterized by peak physical resilience and primary societal productive roles. Data on diet-related IHD mortality and DALYs for this population were obtained from the Global Health Data Exchange Query Tool,^1^ covering 5 Socio-Demographic Index (SDI), 21 GBD regions, and 204 countries and territories from 1990 to 2021. The SDI is a composite indicator with values between 0 and 1 that reflect a country’s or region’s per capita income, education level, and fertility rate under age 25, and is strongly correlated with health outcomes.

### 2.2 Dietary risks

GBD 2021 categorizes risks into three main types: behavioral, environmental and occupational, and metabolic, which are further subdivided into subtypes, individual risks, or groups of risks, with dietary risks being one of the behavioral risks (11). Among GBD 2021, there were 13 dietary risks associated with the outcomes of IHD, including diet low in whole grains, diet low in vegetables, diet low in seafood omega-3 fatty acids, diet low in fruits, diet low in legumes, diet low in nuts and seeds, diet low in polyunsaturated fatty acids and diet low in fiber, and diet high in trans fatty acids, diet high in sugar-sweetened beverages, diet high in sodium, diet high in processed meat and diet high in red meat (11). These authoritative data on dietary risk are derived from a 24-h dietary review survey, which records or converts the daily food and nutrient intake (in grams) per person per day.

### 2.3 Statistical analysis

To quantify the burden of IHD attributable to dietary risks among young adults variables such as mortality rates and DALYs rates were utilized. The methods have been previously described by the GBD studies (12, 13). All estimates were reported per 100,000 person-years with corresponding 95% uncertainty intervals (UIs), derived through GBD computational algorithms. Specifically, 500 computational iterations were performed for each variable across multiple deciles in GBD 2021, with the 2.5th and 97.5th percentiles defining the 95% UI ranges (9). Temporal trends from 1990 to 2021 were analyzed using estimated annual percentage changes (EAPCs) calculated through a semi-log regression model: ln(y) = *α* + *β*x + *ε*, where x represents calendar year, y denotes the outcome rate, α is the intercept, β the slope coefficient, and ε the error term. The EAPC was computed as 100 × (e^β-1). Trend significance was determined by examining the 95% confidence interval (CI) of EAPC: a completely negative CI indicated a significant decreasing trend, a completely positive CI reflected a significant increasing trend, and CI spanning zero denoted non-significant changes.

This study utilized an Autoregressive Integrated Moving Average (ARIMA) model to project the mortality rate, DALYs rate, and corresponding 95% prediction interval (PI) for diet-related IHD among young adults globally during 2022–2031. The ARIMA model is a time-series analysis model where p, d, and q represent the orders of the autoregressive (AR), differencing (I), and moving average (MA) components, respectively. The model was constructed using the auto.arima function from the “forecast” and “tseries” packages, with optimal model selection and parameter determination based on both the Akaike Information Criterion (AIC) and Bayesian Information Criterion (BIC). Model adequacy was verified through Ljung-Box testing to ensure the residuals satisfied the assumptions of independence and normal distribution (14).

All statistical analyses were performed using R software (V.4.3.2).

## 3 Results

### 3.1 Global burden of IHD attributable to dietary risks among young adults from 1990 to 2021

Globally, the mortality rate of diet-related IHD among young people decreased from 10.23 (95%UI: −1.06 to 13.98) in 1990 to 9.48 (95%UI: −1.54 to 13.41) in 2021, with the EAPC of −0.49 (95%CI: −0.61 to −0.37). Similarly, the DALYs rate decreased from 509.63 (95%UI: −55.32 to 687.24) to 465.57 (95%UI: −78.20 to 658.72), with an EAPC of −0.51 (95%CI: −0.62 to −0.39) over the same period. Among 13 dietary risks, the burden of IHD attributable to diets low in whole grains, fruits, and nuts and seeds was the most significant in 2021. Notably, the analysis found that only diets high sugar-sweetened beverages showed an upward trend, with an EAPC of 0.76 (95%CI: 0.72 to 0.80) for mortality rate and 0.78 (95%CI: 0.74 to 0.82) for DALYs rate. In contrast, all other dietary risks demonstrated declining trends. The most substantial decline was observed for high processed meat consumption, with an EAPC of −4.40 (95%CI: −5.10 to −3.69) for mortality rate and −4.38 (95%CI: −5.05 to −3.69) for DALYs rate, whereas the most modest decline was seen for high sodium intake, with an EAPC of −0.12 (95% CI: −0.31 to 0.07) for the mortality rate and −0.15 (95% CI: −0.33 to 0.04) for the DALYs rate (Table 1).

**Table 1:** The global burden of IHD attributable to dietary risks among young adults in 1990 and 2021, along with temporal trends from 1990 to 2021.

Risks	DALYs rate			Mortality rate		
	1990	2021	EAPC	1990	2021	EAPC
Dietary risks	509.63 (−55.32 to 687.24)	465.57 (−78.20 to 658.72)	−0.51 (−0.62 to −0.39)	10.32 (−1.06 to 13.98)	9.48 (−1.54 to 13.41)	−0.49 (−0.61 to −0.37)
Diet low in whole grains	176.04 (117.30 to 227.73)	163.96 (108.46 to 214.21)	−0.49 (−0.62 to −0.36)	3.55 (2.36 to 4.61)	3.32 (2.19 to 4.34)	−0.48 (−0.62 to −0.34)
Diet low in vegetables	50.69 (27.47 to 73.70)	42.43 (22.52 to 62.40)	−0.74 (−0.82 to −0.66)	1.03 (0.55 to 1.49)	0.86 (0.46 to 1.27)	−0.72 (−0.81 to −0.64)
Diet low in seafood omega-3 fatty acids	138.60 (32.81 to 216.10)	105.28 (24.52 to 166.08)	−1.15 (−1.3 to −1.00)	2.79 (0.66 to 4.34)	2.13 (0.49 to 3.35)	−1.13 (−1.28 to −0.97)
Diet low in polyunsaturated fatty acids	107.90 (−442.17 to 388.11)	106.60 (−421.74 to 379.42)	−0.22 (−0.32 to −0.12)	2.18 (−8.89 to 7.86)	2.17 (−8.50 to 7.72)	−0.2 (−0.3 to −0.09)
Diet low in nuts and seeds	139.19 (47.44 to 211.50)	110.67 (36.67 to 168.80)	−1.00 (−1.1 to −0.90)	2.80 (0.95 to 4.27)	2.24 (0.74 to 3.42)	−0.98 (−1.09 to −0.88)
Diet low in legumes	67.73 (−66.55 to 158.32)	53.22 (−48.13 to 124.23)	−1.13 (−1.25 to −1.01)	1.37 (−1.34 to 3.21)	1.08 (−0.97 to 2.54)	−1.11 (−1.23 to −0.99)
Diet low in fruits	137.57 (34.01 to 215.74)	122.01 (30.40 to 189.70)	−0.63 (−0.73 to −0.52)	2.76 (0.68 to 4.34)	2.47 (0.61 to 3.84)	−0.59 (−0.7 to −0.49)
Diet low in fiber	116.51 (67.53 to 159.85)	90.39 (50.92 to 126.19)	−1.08 (−1.28 to −0.89)	2.33 (1.35 to 3.21)	1.82 (1.02 to 2.55)	−1.06 (−1.26 to −0.86)
Diet high in trans fatty acids	31.36 (3.61 to 57.57)	18.02 (1.79 to 34.38)	−2.29 (−2.62 to −1.95)	0.64 (0.07 to 1.18)	0.36 (0.04 to 0.70)	−2.29 (−2.62 to −1.95)
Diet high in sugar-sweetened beverages	1.55 (−0.37 to 3.50)	2.00 (−0.46 to 4.50)	0.78 (0.74 to 0.82)	0.03 (−0.01 to 0.07)	0.04 (−0.01 to 0.09)	0.76 (0.72 to 0.8)
Diet high in sodium	39.73 (9.23 to 100.05)	39.16 (8.06 to 98.16)	−0.15 (−0.33 to 0.04)	0.83 (0.20 to 2.06)	0.82 (0.17 to 2.04)	−0.12 (−0.31 to 0.07)
Diet high in red meat	32.54 (−1.81 to 65.57)	25.96 (−1.45 to 52.22)	−1.00 (−1.19 to −0.81)	0.66 (−0.04 to 1.33)	0.53 (−0.03 to 1.07)	−1.02 (−1.22 to −0.81)
Diet high in processed meat	26.12 (11.28 to 39.04)	10.10 (4.21 to 15.96)	−4.38 (−5.05 to −3.69)	0.54 (0.23 to 0.81)	0.21 (0.09 to 0.33)	−4.4 (−5.1 to −3.69)

### 3.2 Global burden of IHD attributable to dietary risks among young adults by gender in 1990 to 2021

**Figure 1:**
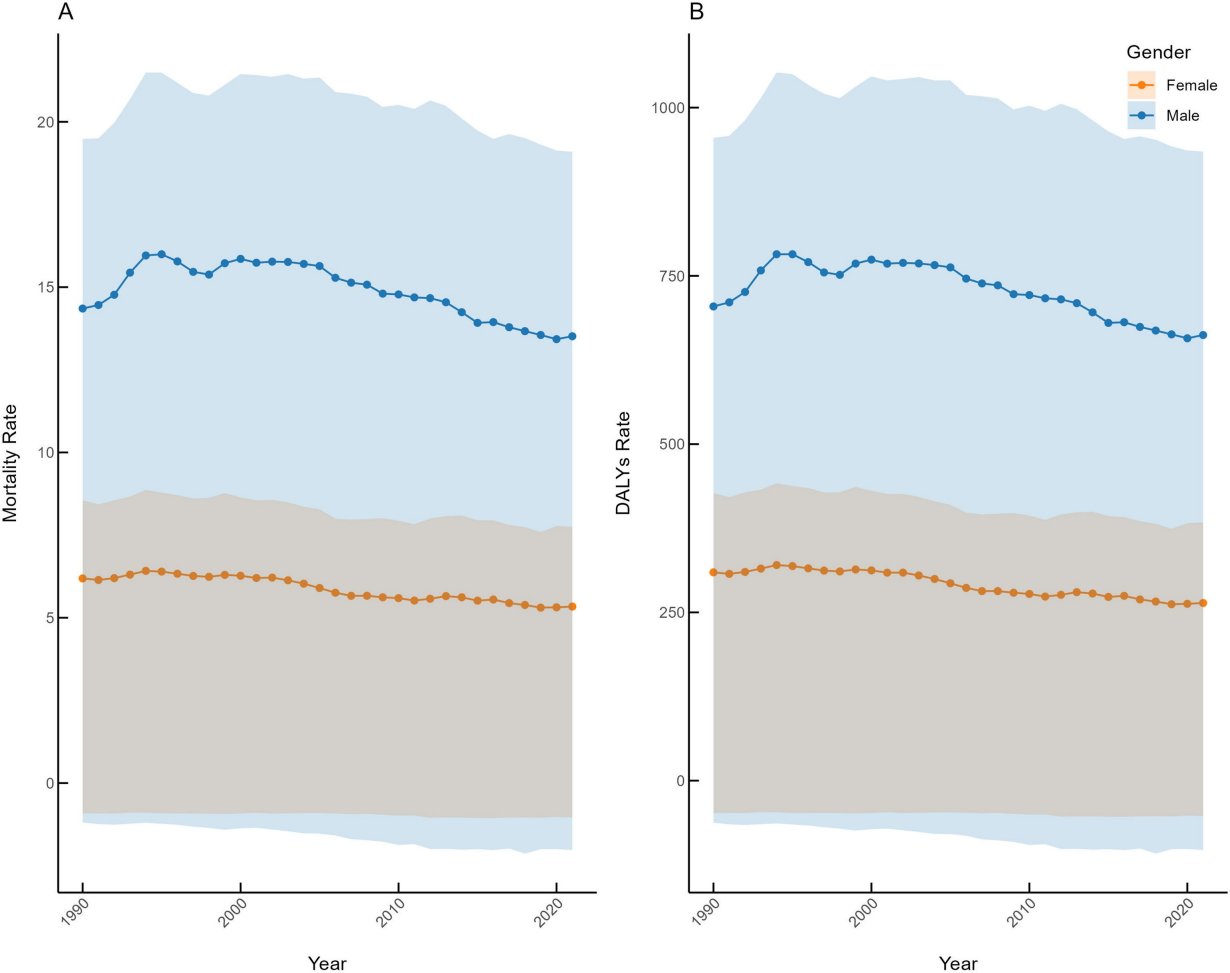
Global gender-specific trends in dietary risk-attributable IHD burden in 1990–2021.

In both 1990 and 2021, the global mortality rate and DALYs rate of IHD attributable to dietary risks were higher among young males than females. From 1990 to 2021, the mortality rate of IHD attributable to dietary risks among young males declined from 14.35 (95% UI: −1.20, 19.48) to 13.51 (95% UI: −2.02, 19.09), with an EAPC of −0.43 (95% CI: −0.57 to −0.28); the dietary-related IHD DALYs rate in males decreased from 704.57 (95% UI: −62.45, 954.97) to 662.08 (95% UI: −103.00, 934.59), with an EAPC of −0.44 (95% CI: −0.58 to −0.30). The decline in both mortality and DALYs rates was more pronounced among females, decreasing from 6.19 (95% UI: −0.92, 8.55) to 5.34 (95% UI: −1.04, 7.75) and from 309.47 (95% UI: −48.00, 427.20) to 263.94 (95% UI: −52.75, 383.80), with corresponding EAPCs of −0.64 (95% CI: −0.72 to −0.55) and −0.68 (95% CI: −0.76 to −0.59), respectively (Figure 1).

### 3.3 The burden of IHD attributable to dietary risks among young adults by 21 GBD regions

In 2021, High-income Asia Pacific exhibited both the lowest mortality rate (2.30; 95% UI: 0.03 to 3.33) and DALY rate (112.43; 95% UI: 1.55 to 160.53), while Eastern Europe showed the highest mortality rate (18.30; 95% UI: −1.50 to 25.54) and the highest DALY rate (890.46; 95% UI: −300.55 to 1426.09). From 1990 to 2021, the steepest decline in mortality and DALY rates occurred in Central Europe, with EAPCs of −3.9 (95% CI: −4.16 to −3.65) and −3.89 (95% CI: −4.13 to −3.65), whereas the most significant increase was observed in East Asia, with EAPCs of 0.58 (95% CI: 0.36 to 0.76) and 0.47 (95% CI: 0.31 to 0.63) (Table 2).

**Table 2:** The burden of IHD attributable to dietary risks among young adults in 21 GBD regions and 5 SDI regions in 1990 and 2021, along with temporal trends observed from 1990 to 2021.

Location	DALYs rate			Mortality rate		
	1990	2021	EAPC	1990	2021	EAPC
High SDI	409.62 (2.43 to 552.42)	240.09 (16.95 to 339.45)	−1.88 (−1.96 to −1.79)	8.49 (0.07 to 11.49)	4.93 (0.37 to 7.01)	−1.91 (−2.02 to −1.81)
High-middle SDI	564.20 (−18.68 to 746.82)	413.37 (−43.18 to 599.54)	−1.83 (−2.26 to −1.41)	11.50 (−0.31 to 15.22)	8.50 (−0.82 to 12.36)	−1.82 (−2.26 to −1.37)
Middle SDI	455.33 (−70.95 to 628.80)	491.36 (−112.45 to 713.45)	0.22 (0.15 to 0.29)	9.11 (−1.35 to 12.61)	9.99 (−2.22 to 14.56)	0.29 (0.22 to 0.36)
Low-middle SDI	664.89 (−115.17 to 906.69)	633.02 (−120.84 to 891.31)	−0.06 (−0.12 to 0)	13.40 (−2.27 to 18.32)	12.85 (−2.40 to 18.10)	−0.02 (−0.08 to 0.04)
Low SDI	412.90 (−55.67 to 580.13)	361.59 (−48.23 to 507.35)	−0.52 (−0.58 to −0.45)	8.43 (−1.12 to 11.85)	7.33 (−0.96 to 10.29)	−0.54 (−0.61 to −0.47)
Andean Latin America	291.41 (−15.93 to 421.35)	195.87 (−28.76 to 300.53)	−1.56 (−1.94 to −1.19)	5.70 (−0.29 to 8.27)	3.81 (−0.55 to 5.89)	−1.59 (−1.97 to −1.21)
Australasia	337.59 (−114.36 to 483.75)	135.82 (−43.86 to 201.46)	−3.06 (−3.24 to −2.88)	7.03 (−2.36 to 10.11)	2.81 (−0.89 to 4.17)	−3.07 (−3.25 to −2.89)
Caribbean	467.57 (−16.36 to 661.90)	410.87 (53.23 to 620.33)	−0.37 (−0.62 to −0.12)	9.51 (−0.34 to 13.54)	8.39 (1.09 to 12.74)	−0.34 (−0.59 to −0.10)
Central Asia	936.10 (−65.45 to 1185.33)	682.71 (−91.83 to 930.10)	−2.15 (−2.72 to −1.58)	18.92 (−1.19 to 23.95)	13.93 (−1.83 to 19.06)	−2.09 (−2.68 to −1.49)
Central Europe	1030.22 (−0.57 to 1318.54)	378.87 (5.66 to 521.10)	−3.89 (−4.13 to −3.65)	21.35 (0.08 to 27.36)	7.92 (0.16 to 10.94)	−3.9 (−4.16 to −3.65)
Central Latin America	300.04 (−34.09 to 418.78)	338.17 (−39.96 to 503.99)	0.24 (−0.1 to 0.57)	5.97 (−0.66 to 8.36)	6.81 (−0.78 to 10.21)	0.25 (−0.08 to 0.58)
Central Sub-Saharan Africa	234.56 (−83.98 to 409.50)	231.53 (−66.79 to 388.28)	−0.21 (−0.3 to −0.13)	4.86 (−1.74 to 8.50)	4.78 (−1.37 to 8.07)	−0.22 (−0.30 to −0.13)
East Asia	307.35 (−20.73 to 429.88)	347.30 (−31.35 to 535.54)	0.47 (0.31 to 0.63)	6.10 (−0.36 to 8.53)	7.03 (−0.57 to 10.85)	0.58 (0.39 to 0.76)
Eastern Europe	1147.25 (41.67 to 1452.28)	873.09 (−75.46 to 1216.55)	−2.41 (−3.22 to −1.59)	23.78 (0.92 to 30.13)	18.30 (−1.50 to 25.54)	−2.39 (−3.23 to −1.54)
Eastern Sub-Saharan Africa	213.50 (−23.17 to 313.97)	191.69 (−51.81 to 291.45)	−0.66 (−0.76 to −0.55)	4.29 (−0.44 to 6.33)	3.81 (−1.02 to 5.82)	−0.71 (−0.82 to −0.59)
High-income Asia Pacific	161.35 (−19.25 to 241.40)	112.43 (1.55 to 160.53)	−1.18 (−1.39 to −0.98)	3.26 (−0.36 to 4.89)	2.30 (0.03 to 3.33)	−1.13 (−1.33 to −0.94)
High-income North America	437.49 (92.68 to 579.54)	259.00 (95.91 to 339.58)	−1.83 (−2.15 to −1.52)	9.13 (1.95 to 12.10)	5.38 (1.99 to 7.07)	−1.84 (−2.18 to −1.5)
North Africa and Middle East	888.75 (−102.20 to 1239.90)	686.20 (−82.16 to 1024.42)	−0.90 (−1.02 to −0.77)	17.88 (−2.04 to 24.99)	13.92 (−1.66 to 20.82)	−0.89 (−1.01 to −0.76)
Oceania	856.02 (−180.31 to 1297.95)	890.46 (−300.55 to 1426.09)	0.21 (0.12 to 0.30)	17.61 (−3.64 to 26.73)	18.26 (−6.14 to 29.27)	0.21 (0.13 to 0.30)
South Asia	763.18 (−131.70 to 1037.31)	759.41 (−113.34 to 1041.63)	0.07 (−0.03 to 0.16)	15.43 (−2.60 to 20.99)	15.46 (−2.25 to 21.27)	0.10 (0.01 to 0.20)
Southeast Asia	466.94 (−230.40 to 710.64)	471.29 (−345.01 to 798.26)	0.09 (0.02 to 0.15)	9.28 (−4.45 to 14.13)	9.58 (−6.85 to 16.22)	0.18 (0.11 to 0.25)
Southern Latin America	452.93 (5.49 to 579.73)	188.29 (3.04 to 251.11)	−2.75 (−3.00 to −2.51)	9.39 (0.14 to 12.07)	3.86 (0.07 to 5.17)	−2.79 (−3.03 to −2.56)
Southern Sub-Saharan Africa	338.22 (−82.68 to 479.80)	290.57 (−10.22 to 407.54)	−0.59 (−1.06 to −0.12)	6.76 (−1.63 to 9.59)	5.91 (−0.19 to 8.30)	−0.53 (−0.97 to −0.09)
Tropical Latin America	445.72 (162.30 to 600.84)	281.30 (75.83 to 402.24)	−1.67 (−1.80 to −1.55)	9.07 (3.29 to 12.25)	5.73 (1.52 to 8.21)	−1.68 (−1.79 to −1.57)
Western Europe	371.27 (−32.30 to 508.00)	134.04 (1.94 to 183.23)	−3.23 (−3.36 to −3.1)	7.71 (−0.66 to 10.60)	2.76 (0.04 to 3.78)	−3.25 (−3.38 to −3.11)
Western Sub-Saharan Africa	187.61 (−69.83 to 295.27)	163.76 (−83.15 to 262.88)	−0.45 (−0.60 to −0.29)	3.85 (−1.42 to 6.09)	3.32 (−1.68 to 5.39)	−0.49 (−0.65 to −0.33)

### 3.4 Burden of SDI values on IHD burden attributable to dietary risks among young adults

**Figure 2:**
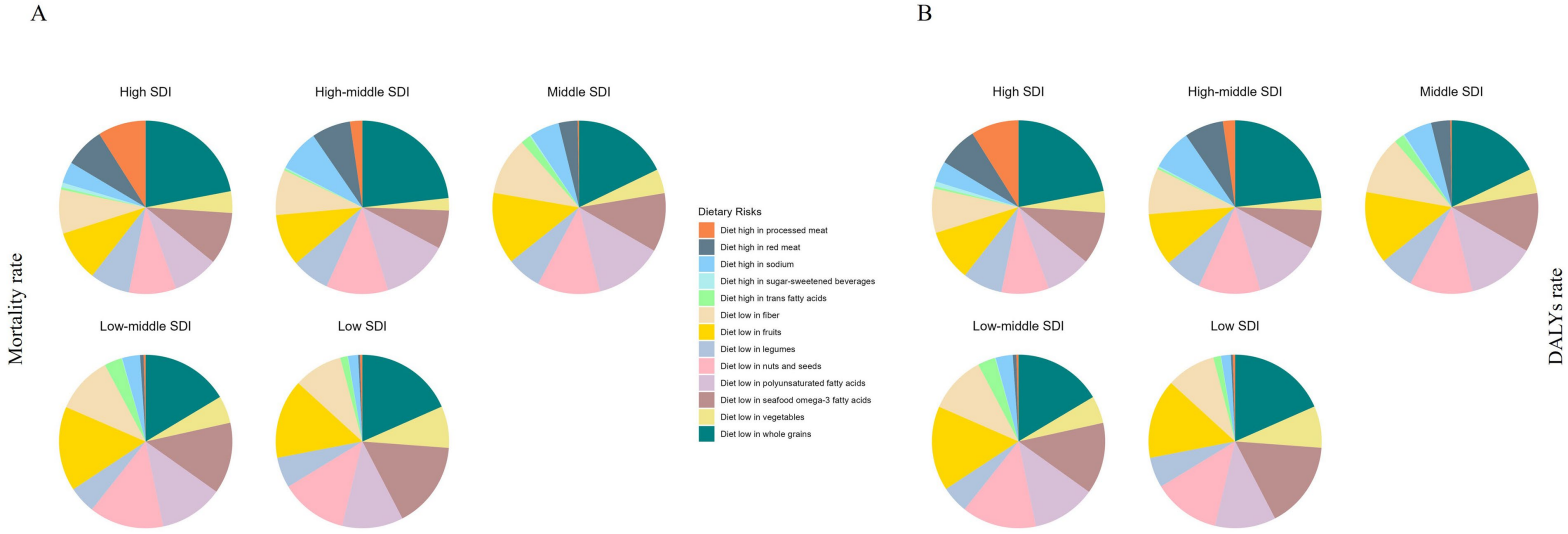
Proportion of IHD mortality rate **(A)** and DALYs rate **(B)** attributable to 13 dietary risks among young adults, for 5 SDI in 2021.

In 2021, regions with low-middle SDI showed the highest mortality rate (12.85, 95%UI: −2.40 to 18.10) and DALYs rate (633.02, 95%UI: −120.84 to 891.31) for IHD attributable to dietary risks. Conversely, the lowest mortality rate (4.93, 95%UI: 0.37 to 7.01) and DALYs rate (240.09, 95%UI: 16.95 to 339.45) were observed in the high SDI regions (Table 2). Additionally, the analysis found that the 5 SDI regions differing proportions of 13 dietary risks in 2021. In terms of both mortality and DALYs rates, diet low in whole grains accounted for the highest proportion in all 5 SDI regions, while diet high in sugar-sweetened beverages accounted for the lowest proportion, except for the middle SDI regions (Figure 2).

**Figure 3:**
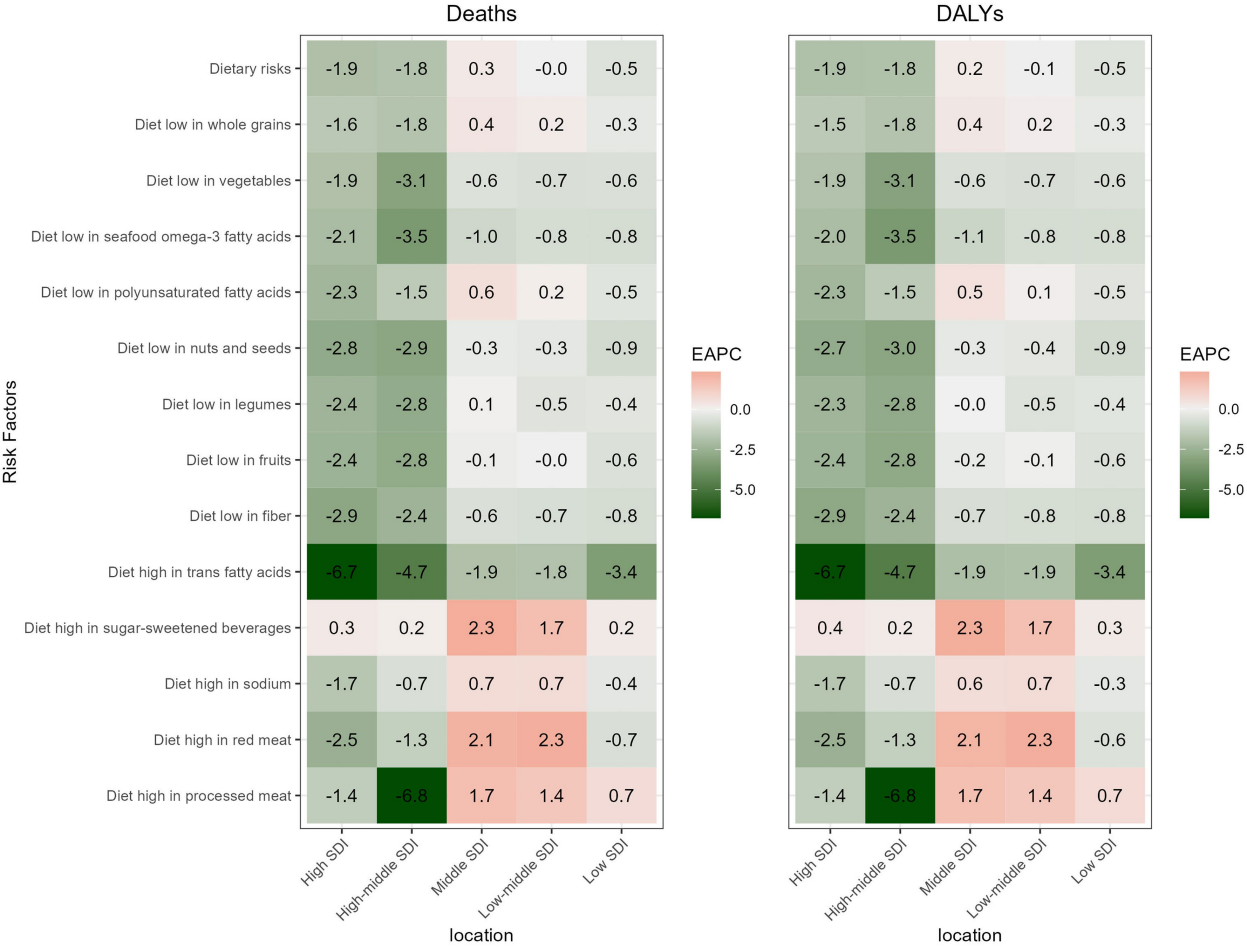
The EAPC of IHD attributable to 13 dietary risks among young adults by SDI regions from 1990 to 2021.

From 1990 to 2021, high SDI regions exhibited the largest decreasing trend in both mortality rate (EAPC: −1.91, −2.02 to −1.81), and DALYs rate (EAPC: −1.88, −1.96 to −1.79). Conversely, the only upward trend in mortality rate (EAPC: 0.29, 0.22 to 0.36) and DALYs rate (EAPC: 0.22, 0.15 to 0.29) was observed in the middle SDI regions (Table 2). Similarly, the mortality rate and DALYs rate associated with 13 dietary factors showed different trends across the 5 SDI regions. Firstly, a diet high in sugar-sweetened beverages showed an increasing trend, while diets low in nuts and seeds, seafood omega-3 fatty acids, fruits, fiber, vegetables and diet high in trans fatty acids exhibited a decline trend in terms of both mortality rate and DALYs rate, across all 5 SDI regions. Additionally, for mortality rate and DALYs rate, diet high in processed meat demonstrated an upward trend in low, low-middle and middle SDI regions. Meanwhile, diets high in red meat and salt, low in whole grain and low in polyunsaturated fatty acid showed an upward trend in low-middle and middle SDI regions. Differently, the mortality rate associated with a low legume diet was on the rise in the middle SDI regions, whereas its DALYs rate remained stable (Figure 3).

### 3.5 The burden of IHD attributable to dietary risks among young adults by country

**Figure 4:**
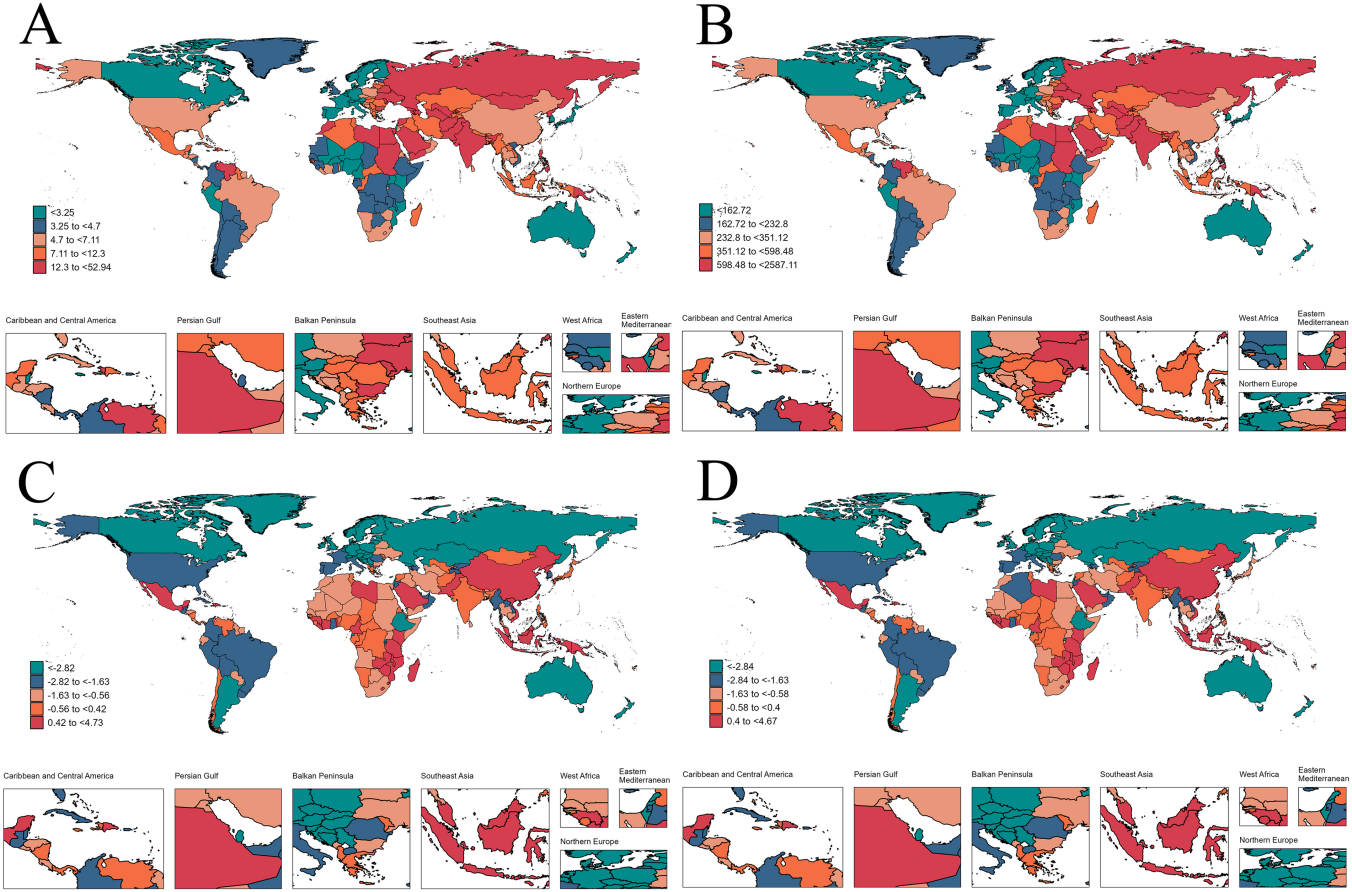
The mortality rate **(A)** and DALYs rate **(B)** in 2021, and the EAPC of the mortality rate **(C)** and DALYs rate **(D)** from 1990 to 2021 of IHD attributable to dietary risks among young adults, by country.

In 2021, Nauru exhibited both the highest mortality rate (52.94, 95%UI: −22.09 to 93.00) and DALYs rate (2587.11, 95%UI: −1074.20 to 4542.85). On the contrary, the lowest mortality rates (1.33, 95%UI: −0.16 to 1.92) and DALYs rate (67.81, 95%UI: −8.02 to 97.74) were found in Sweden. From 1990 to 2021, the largest increases in mortality rate and DALYs rate were observed in Zimbabwe, with EAPCs of 4.73 (95%CI: 3.76 to 5.70) and 4.67 (95%CI: 3.26 to 5.63). In contrast, the most significant declines in mortality rate and DALYs rate were exhibited by Estonia, with EAPCs of −6.97 (95%CI-7.52 to −6.42) and-6.89 (95%CI: −7.42 to −6.36) (Figure 4).

### 3.6 Projections of IHD attributable to dietary risks among young adults, 2022–2031

**Figure 5:**
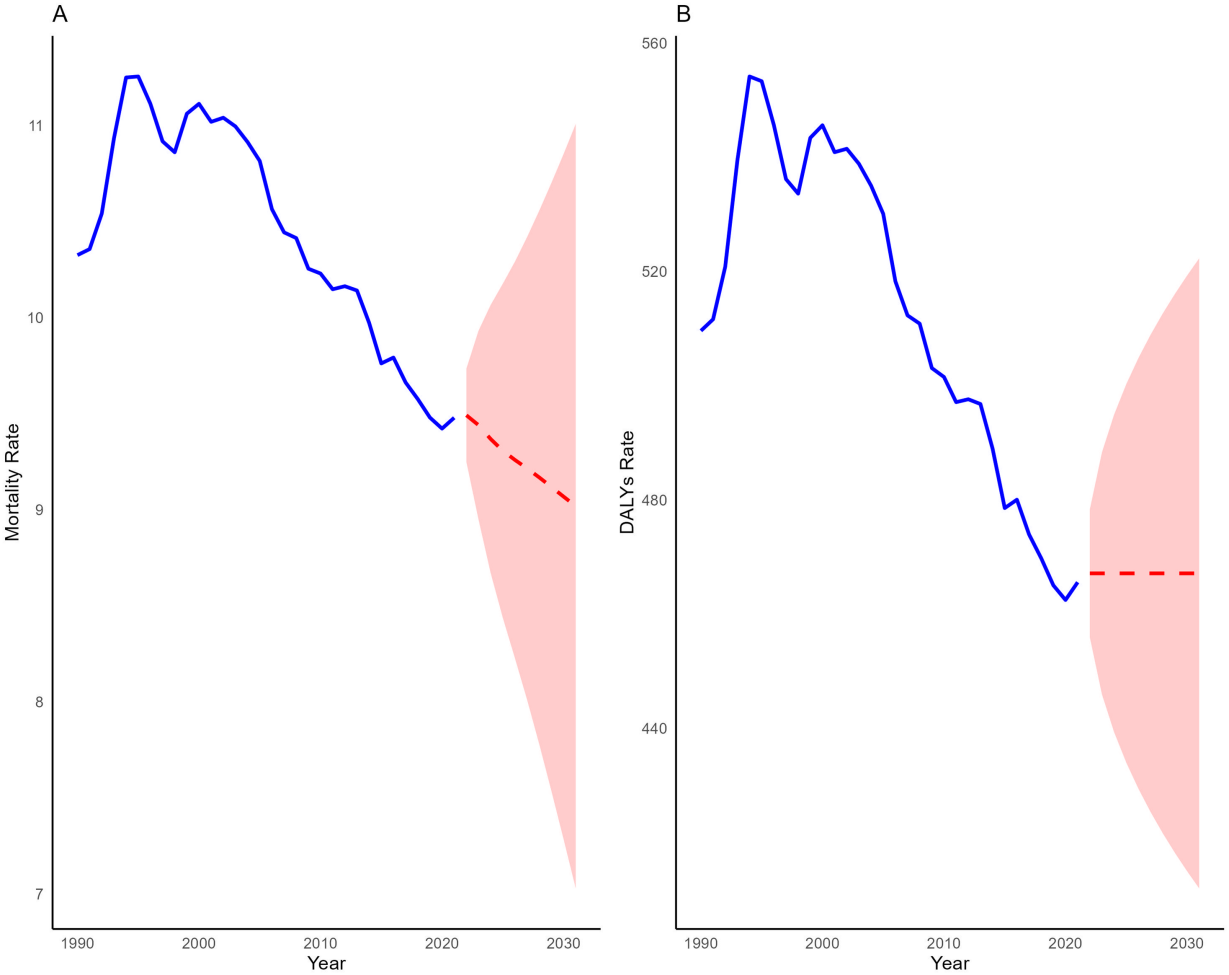
Predicted global trends of IHD attributable to dietary risks among young adults over the next 10 years.

The ARIMA model projects that over the next decade, the mortality rate of IHD attributable to dietary risks among young people will continue to decline, while the DALYs rate is expected to stabilize. By 2031, the mortality rate is projected to decrease to 9.02 (95% PI: 7.02 to 11.00), reflecting a 4.85% reduction compared to 2021, whereas the DALYs rate is anticipated to show a slight increase of 0.34%, reaching 467.13 (95% PI: 411.94 to 522.32) (Figure 5).

## 4 Discussion

Our analysis of the GBD data is the first to provide a comprehensive overview of the global, regional, and national burden of IHD attributable to dietary risks among young adults from 1990 to 2021. Over the past few decades, the global mortality rate and DALYs rate of IHD among young people attributed to dietary risks have shown a significantly decline. This trend is not only closely related to the improvement of living standards and the enhancement of self-health awareness (15), but also to the countries’ proactive responses to WHO initiatives, the development of nutrition guidelines tailored to local residents, and increased promotion of healthy diets (16). However, a notable finding from our study is that only high sugar beverage diet related IHD showed an increasing trend among young adults. This finding contrasts sharply with the overall decline observed in cardiovascular disease attributed to a high sugar beverage diet across the entire population (17). This may be closely associated with the consumption habits of young individuals, who regularly consume high sugar-sweetened beverages in their diets. In the United States, approximately 31% of adults consume sugar-sweetened beverages at least once a day, but most adults are unaware of the beverages’ actual calorie content, potentially influenced by their educational attainment (18). Consequently, it is crucial for the food and beverage industry to accelerate product reformulation to reduce added sugar and intensify the development of healthier, low- or no-sugar beverage alternatives.

At the regional level, SDI serves as a key indicator to measure the socio-economic development of a country or region. In this study, we observed significant regional heterogeneity in IHD among adults aged 25–49 caused by dietary risk factors. In this study, we identified three key aspects of regional heterogeneity in the incidence of IHD among young adults caused by dietary risk factors:

Firstly, the highest burden of IHD mortality rate and DALYs rate in 2021 was observed in the low-middle SDI regions, while the high SDI regions had the lowest mortality rate and DALYs rate. This is consistent with the global disease burden of IHD attributed to dietary factors in the general population (19). It is clear that compared to high SDI regions, low SDI or low-middle SDI regions experienced a heavier burden due to economic conditions, significantly insufficient healthcare investment, and limited access advanced medical technology and facilities (20). However, from 1990 to 2021, only middle SDI regions exhibited an upward trend in IHD attributable to dietary risks, contrary to the declining trend observed in the general population (19), which likely reflects the complex interplay of socio-cultural influences, demographic structure, and region-specific processes such as rapid urbanization and nutritional transition. This phase is characterized by increased consumption of processed foods and sugar-sweetened beverages alongside persistent deficits in protective foods, against a backdrop of healthcare systems that are still developing capacity for chronic disease prevention and management (21, 22). Therefore, young adults in middle SDI regions should give greater attention to diet-related cardiovascular health to mitigate the risk of IHD.

Secondly, it is worth noting that there were certain differences in the performance of 13 dietary risks among the 5 SDI regions. This study identified that the risks of IHD attributed to high processed and red meat diets among adults was more significant in high SDI and high-middle SDI regions in 2021. This finding aligns with the global burden of IHD attributed to high red meat and processed meat diets throughout all age groups (23). On the contrary, IHD related to diets low in nuts and seeds, seafood omega-3 fatty acids, fruits, fiber, and vegetables was subject to a greater burden in low, low-middle and middle SDI regions. As pointed out by relevant research, groups with poorer socio-economic conditions often exhibit lower intake of seafood, nuts and seeds, fruits, vegetables, and dietary fiber (24–26). In high SDI regions, the gradual improvement in residents’ dietary structures is driven by economic development, increased awareness of healthy eating, and the active implementation of policies and regulations. Residents in these areas have increasingly focused on consuming healthier foods, such as whole grains, vegetables, and fruits, while reducing their intake of high-risk foods, including salt, processed meat, and red meat (27, 28). In contrast, in the middle and low SDI regions, although economic development has led to an improvement in living standards, residents in these areas experience ongoing challenges from unhealthy eating habits such as high salt and high fat consumption, due to the acceleration of nutrition transformation, insufficient popularization of health education, and the promotion of urbanization. The high intake of processed meat and red meat, as well as insufficient intake of healthy foods such as whole grains, vegetables, and fruits, remains a growing concern (29). Therefore, the food industry should prioritize reformulating processed and red meat products and clearly label these improvements to guide consumer choice in high SDI regions, while focusing on enhancing the palatability, convenience, and affordability of healthy, culturally appropriate foods in middle- and low-SDI regions to bridge the intake gap. Moreover, a distinctive pattern was observed in middle SDI regions, where IHD mortality rate associated with low legume intake showed an increasing trend while the DALYs rate remained stable. This pattern reflected the ongoing healthcare transition in these regions: while enhanced acute cardiac care systems have reduced acute event mortality, the lack of chronic disease management has led to long-term illness survival among survivors (30). The consequent increase in years lived with disability (YLDs) offsets the reduction in years of life lost (YLLs), resulting in stabilized DALYs. Concurrently, low legume intake exacerbates metabolic disorders and chronic inflammation through multiple mechanisms—including insufficient dietary fiber, inadequate plant protein, and deficient antioxidant intake—and synergizes with high-salt, high-fat dietary patterns during nutritional transition to promote IHD risk, ultimately manifesting as rising mortality (31). This phenomenon highlights the imbalance between acute and chronic care in healthcare systems and underscores the profound impact of dietary pattern changes on cardiovascular health.

Despite regional variations, the burden of diet low in whole grains was consistently most pronounced in all 5 SDI regions. While whole grain diets are a relatively new dietary concept, many countries have incorporated whole grains into their dietary guidelines, the population adherence to these guidelines remains generally poor. According to a systematic evaluation, 50.5% of Germans consume whole grains, and less than 40% of the population in low- and middle-income countries reach the recommendation of adequate grain intake (32). According to a survey conducted in South Africa, it was found that 36% of respondents knew nothing or very little about whole grains (33). A whole grain diet may reduce cardiometabolic risk and inflammatory responses, thereby lowering the burden of IHD (34, 35). Therefore, regions should not only enhance public awareness of the health benefits of whole grains but also actively encourage the food industry to invest in innovation for enhancing product palatability and developing affordable, convenient whole-grain products, thereby ensuring that healthier choices are both accessible and appealing.

In terms of gender, the study also revealed that the IHD mortality rate and DALYs rate attributed to dietary risk among young adults, with males consistently exhibiting higher levels than females, consistent with the overall burden of IHD disease in the population (36). A review analysis on gender differences in heart injury shows that women have better myocardial contraction function and better ability to recover from heart ischemia compared to men of the same age group, and the presence of estrogen is thought to enhance women’s heart tolerance to injury (37). Furthermore, women are generally more inclined to consume foods that are rich in fiber and low in fat. On the other hand, men may be more likely to opt for a diet that is high in fat and calories (38). Therefore, young individuals, particularly men, should increase their intake of whole grains, fruits, nuts, and seeds, while decreasing the consumption of processed and red meat, in order to adopt a more balanced diet.

Disease burden projections serve as a critical scientific foundation for public health policymaking. Over the next decade, the global mortality rate of IHD among young adults is projected to decline by 4.85%, while the DALYs rate is expected to increase slightly by 0.34% and remain relatively stable. This trend likely reflects an epidemiological shift—improvements in acute-phase care have reduced case fatality rates, while insufficient chronic disease management has led to accumulating disability among survivors (39). These findings suggest that future IHD prevention and control should adopt a dual-track strategy: maintaining existing acute care systems while prioritizing rehabilitation services and chronic disease management, particularly in low- and middle-income countries, to achieve comprehensive burden reduction.

## 5 Limitations

This study has several limitations. First, although the GBD 2021 employs a comparative risk assessment framework, residual confounding from covariates such as smoking and physical activity may affect the estimation of independent effects of dietary risks. Second, the dietary exposure data, derived from 24-h recalls, lack detail on food preparation methods and types of cooking oils used. This likely leads to exposure misclassification, as different oils (e.g., olive oil vs. partially hydrogenated oils) have divergent effects on IHD risk, thereby introducing bias into burden estimates. Third, the analysis was restricted to adults aged 25–49 years due to the lack of GBD estimates for the 15–24 age group, limiting insights into the burden among younger adults.

## 6 Conclusion

Although the burden of IHD attributable to dietary risks among young individuals has decreased in recent years, the disease burden of IHD remains heavy in low, low- middle, and middle SDI regions. Through systematic analysis of dietary risks for IHD in young adults during 1990–2021 and 10-year burden projections (2022–2031), this study comprehensively evaluated the population attributable fractions of 13 dietary risks and identified region-specific predominant dietary determinants. These evidence-based results provide a robust scientific foundation for developing targeted, precision dietary interventions tailored to specific populations across different regions.

## Data Availability

The original contributions presented in the study are included in the article/Supplementary material, further inquiries can be directed to the corresponding authors.

## Footnotes

1
http://ghdx.healthdata.org/gbd-results-tool

## References

[1] Jensen RV Hjortbak MV Bøtker HE. Ischemic heart disease: an update. Semin Nucl Med. (2020) 50:195–207. doi: 10.1053/j.semnuclmed.2020.02.007, 32284106

[2] Sagris M Antonopoulos AS Theofilis P Oikonomou E Siasos G Tsalamandris S . Risk factors profile of young and older patients with myocardial infarction. Cardiovasc Res. (2022) 118:2281–92. doi: 10.1093/cvr/cvab264, 34358302

[3] Chen S-M Tsai T-H Hang C-L Yip H-K Fang C-Y Wu C-J . Endothelial dysfunction in young patients with acute ST-elevation myocardial infarction. Heart Vessel. (2011) 26:2–9. doi: 10.1007/s00380-010-0017-0, 20949355

[4] World Health Organization. The top 10 causes of death. Available online at: https://www.who.int/news-room/fact-sheets/detail/the-top-10-causes-of-death (August 7, 2024)

[5] Li X Jiang H. Global, regional, and national burden of ischaemic heart disease and its attributable risk factors in youth from 1990 to 2019: a global burden of disease study. Public Health. (2024) 236:43–51. doi: 10.1016/j.puhe.2024.07.011, 39159577

[6] Wu P Yu S Wang J Zou S Yao D-S Xiaochen Y. Global burden, trends, and inequalities of ischemic heart disease among young adults from 1990 to 2019: a population-based study. Front Cardiovasc Med. (2023) 10:1274663. doi: 10.3389/fcvm.2023.1274663, 38075966 PMC10704897

[7] Mozaffarian D. Dietary and policy priorities for cardiovascular disease, diabetes, and obesity: a comprehensive review. Circulation. (2016) 133:187–225. doi: 10.1161/CIRCULATIONAHA.115.018585, 26746178 PMC4814348

[8] Appleton KM. Liking for sweet taste, sweet food intakes, and sugar intakes. Nutrients. (2024) 16:3672. doi: 10.3390/nu16213672, 39519505 PMC11547215

[9] GBD 2021 Diseases and Injuries Collaborators. Global incidence, prevalence, years lived with disability (YLDs), disability-adjusted life-years (DALYs), and healthy life expectancy (HALE) for 371 diseases and injuries in 204 countries and territories and 811 subnational locations, 1990-2021: a systematic analysis for the global burden of disease study 2021. Lancet. (2024) 403:2133–61. doi: 10.1016/S0140-6736(24)00757-8 38642570 PMC11122111

[10] GBD 2021 Risk Factors Collaborators. Global burden and strength of evidence for 88 risk factors in 204 countries and 811 subnational locations, 1990-2021: a systematic analysis for the global burden of disease study 2021. Lancet. (2024) 403:2162–203. doi: 10.1016/S0140-6736(24)00933-4 38762324 PMC11120204

[11] GHDx (2024). Global Burden of Disease Study 2021 (GBD 2021) Data Resources. Available online at: https://ghdx.healthdata.org/gbd-2021 (Accessed September 19, 2024)

[12] Safiri S Nejadghaderi SA Karamzad N Carson-Chahhoud K Bragazzi NL Sullman MJM . Global, regional, and national cancer deaths and disability-adjusted life-years (DALYs) attributable to alcohol consumption in 204 countries and territories, 1990-2019. Cancer. (2022) 128:1840–52. doi: 10.1002/cncr.34111, 35239973

[13] Murray CJ Acharya AK. Understanding DALYs (disability-adjusted life years). J Health Econ. (1997) 16:703–30. doi: 10.1016/s0167-6296(97)00004-0, 10176780

[14] Zheng A Fang Q Zhu Y Jiang C Jin F Wang X. An application of ARIMA model for predicting total health expenditure in China from 1978-2022. J Glob Health. (2020) 10:010803. doi: 10.7189/jogh.10.010803, 32257167 PMC7101215

[15] Kaczorowski J Chambers LW Dolovich L Paterson JM Karwalajtys T Gierman T . Improving cardiovascular health at population level: 39 community cluster randomised trial of cardiovascular health awareness program (CHAP). BMJ. (2011) 342:d442. doi: 10.1136/bmj.d442, 21300712 PMC3034422

[16] Springmann M Spajic L Clark MA Poore J Herforth A Webb P . The healthiness and sustainability of national and global food based dietary guidelines: modelling study. BMJ. (2020) 370:m2322. doi: 10.1136/bmj.m2322, 32669369 PMC7362232

[17] Shi D Tao Y Wei L Yan D Liang H Zhang J . The burden of cardiovascular diseases attributed to diet high in sugar-sweetened beverages in 204 countries and territories from 1990 to 2019. Curr Probl Cardiol. (2024) 49:102043. doi: 10.1016/j.cpcardiol.2023.102043, 37595857

[18] Park S Onufrak S Sherry B Blanck HM. The relationship between health-related knowledge and sugar-sweetened beverage intake among US adults. J Acad Nutr Diet. (2014) 114:1059–66. doi: 10.1016/j.jand.2013.11.003, 24360502 PMC4470487

[19] Rostami R Moradinazar M Moradi S Samannejad B Cheshmeh S Saber A . Impact of dietary risk on global ischemic heart disease: findings from 1990–2019. Sci Rep. (2024) 14:18012. doi: 10.1038/s41598-024-69089-w, 39097603 PMC11297957

[20] Rosengren A Smyth A Rangarajan S Ramasundarahettige C Bangdiwala SI AlHabib KF . Socioeconomic status and risk of cardiovascular disease in 20 low-income, middle-income, and high-income countries: the prospective urban rural epidemiologic (PURE) study. Lancet Glob Health. (2019) 7:e748–60. doi: 10.1016/S2214-109X(19)30045-2, 31028013

[21] Parajára MC Colombet Z Machado ÍE Menezes MC Verly-Jr E O’Flaherty M . Mortality attributable to diets low in fruits, vegetables, and whole grains in Brazil in 2019: evidencing regional health inequalities. Public Health. (2023) 224:123–30. doi: 10.1016/j.puhe.2023.08.028, 37774566

[22] Mente A Dehghan M Rangarajan S O’Donnell M Hu W Dagenais G . Diet, cardiovascular disease, and mortality in 80 countries. Eur Heart J. (2023) 44:2560–79. doi: 10.1093/eurheartj/ehad269 37414411 PMC10361015

[23] Yan D Liu K Li F Shi D Wei L Zhang J . Global burden of ischemic heart disease associated with high red and processed meat consumption: an analysis of 204 countries and territories between 1990 and 2019. BMC Public Health. (2023) 23:2267. doi: 10.1186/s12889-023-16954-4, 37978363 PMC10655305

[24] de Souza RJ Dehghan M Mente A Bangdiwala SI Ahmed SH Alhabib KF . Association of nut intake with risk factors, cardiovascular disease, and mortality in 16 countries from 5 continents: analysis from the prospective urban and rural epidemiology (PURE) study. Am J Clin Nutr. (2020) 112:208–19. doi: 10.1093/ajcn/nqaa108, 32433740

[25] Rahman MN Islam ARMT. Consumer fish consumption preferences and contributing factors: empirical evidence from Rangpur city corporation, Bangladesh. Heliyon. (2020) 6:e05864. doi: 10.1016/j.heliyon.2020.e05864, 33426347 PMC7779775

[26] Giskes K Avendano M Brug J Kunst AE. A systematic review of studies on socioeconomic inequalities in dietary intakes associated with weight gain and overweight/obesity conducted among European adults. Obes Rev. (2010) 11:413–29. doi: 10.1111/j.1467-789X.2009.00658.x, 19889178

[27] Jo G Park D Lee J Kim R Subramanian SV Oh H . Trends in diet quality and Cardiometabolic risk factors among Korean adults, 2007-2018. JAMA Netw Open. (2022) 5:e2218297. doi: 10.1001/jamanetworkopen.2022.18297, 35731513 PMC9218851

[28] Alae-Carew C Green R Stewart C Cook B Dangour AD Scheelbeek PFD. The role of plant-based alternative foods in sustainable and healthy food systems: consumption trends in the UK. Sci Total Environ. (2022) 807:151041. doi: 10.1016/j.scitotenv.2021.151041, 34673070 PMC8724617

[29] Popkin BM Adair LS Ng SW. Global nutrition transition and the pandemic of obesity in developing countries. Nutr Rev. (2012) 70:3–21. doi: 10.1111/j.1753-4887.2011.00456.x, 22221213 PMC3257829

[30] Kruk ME Gage AD Arsenault C Jordan K Leslie HH Roder-DeWan S . High-quality health systems in the sustainable development goals era: time for a revolution. Lancet Glob Health. (2018) 6:e1196–252. doi: 10.1016/S2214-109X(18)30386-3, 30196093 PMC7734391

[31] Afshin A Micha R Khatibzadeh S Mozaffarian D. Consumption of nuts and legumes and risk of incident ischemic heart disease, stroke, and diabetes: a systematic review and meta-analysis. Am J Clin Nutr. (2014) 100:278–88. doi: 10.3945/ajcn.113.076901, 24898241 PMC4144102

[32] Leme ACB Hou S Fisberg RM Fisberg M Haines J. Adherence to food-based dietary guidelines: a systemic review of high-income and low- and middle-income countries. Nutrients. (2021) 13:1038. doi: 10.3390/nu13031038, 33807053 PMC8004702

[33] Taylor JRN Rehm CD de Kock HL Donoghue S Johnson A Thompson C . South African consumers’ knowledge, opinions and awareness of whole grains and their health benefits: a Cross-sectional online survey. Nutrients. (2023) 15:3522. doi: 10.3390/nu15163522, 37630713 PMC10457809

[34] Hollænder PLB Ross AB Kristensen M. Whole-grain and blood lipid changes in apparently healthy adults: a systematic review and meta-analysis of randomized controlled studies. Am J Clin Nutr. (2015) 102:556–72. doi: 10.3945/ajcn.115.109165, 26269373

[35] Liu H Zhu J Gao R Ding L Yang Y Zhao W . Estimating effects of whole grain consumption on type 2 diabetes, colorectal cancer and cardiovascular disease: a burden of proof study. Nutr J. (2024) 23:49. doi: 10.1186/s12937-024-00957-x, 38741117 PMC11092208

[36] Guan C Wu S Xu W Zhang J. Global, regional, and national burden of ischaemic heart disease and its trends, 1990-2019. Public Health. (2023) 223:57–66. doi: 10.1016/j.puhe.2023.07.010, 37604031

[37] Ostadal B Ostadal P. Sex-based differences in cardiac ischaemic injury and protection: therapeutic implications. Br J Pharmacol. (2014) 171:541–54. doi: 10.1111/bph.12270, 23750471 PMC3969071

[38] Westenhoefer J. Age and gender dependent profile of food choice. Forum Nutr. (2005). 44–51. doi: 10.1159/000083753 15702587

[39] Teo KK Rafiq T. Cardiovascular risk factors and prevention: a perspective from developing countries. Can J Cardiol. (2021) 37:733–43. doi: 10.1016/j.cjca.2021.02.009, 33610690

