# Supplementary material for: Global burden of ischemic heart disease attributable to dietary risks in young adults, 1990–2021: trends and future projections
Source: Front Public Health. 2026 Feb 13;14:1729653. doi: 10.3389/fpubh.2026.1729653 (PMC12945797; doi:10.3389/fpubh.2026.1729653)
Supplement: Supplementary file 1 [file Table_1.DOCX]

SUPPLEMENTAL MATERIAL

**Table S1** Data on IHD attributable to dietary risks among adults aged 25-49 years in 2021 by gender and age.

| DALYs |  |  |  |
| --- | --- | --- | --- |
| Age |  | Number | Rate |
| 25 to 49 | Male | 13239794.78  (-2059805.22 to 18689168.13) | 662.08  (-103.00 to 934.59) |
|  | Female | 5143794.29  (-1028041.87 to 7479743.54) | 263.94  (-52.75 to 383.80) |
| 25 to 29 | Male | 818015.10  (-221027.68 to 1161037.42) | 275.10  (-74.33 to 390.45) |
|  | Female | 394968.13  (-97240.93 to 566384.89) | 135.73  (-33.42 to 194.64) |
| 30 to 34 | Male | 1607662.71  (-351253.75 to 2264720.76) | 526.15  (-114.96 to 741.20) |
|  | Female | 641818.05  (-155709.04 to 922267.78) | 214.70  (-52.09 to 308.52) |
| 35 to 39 | Male | 2354232.92  (-451254.95 to 3316762.09) | 831.70  (-159.42 to 1171.74) |
|  | Female | 880189.27  (-198256.27 to 1272442.87) | 316.84  (-71.37 to 458.04) |
| 40 to 44 | Male | 3660389.08  (-555304.02 to 5187697.00) | 1451.61  (-220.22 to 2057.30) |
|  | Female | 1337952.47  (-276412.15 to 1926442.22) | 539.30  (-111.42 to 776.51) |
| 45 to 49 | Male | 4799494.97  (-496083.80 to 6839585.64) | 2017.76  (-208.56 to 2875.44) |
|  | Female | 1888866.37  (-300423.48 to 2760920.16) | 801.58  (-127.49 to 1171.66) |
| Mortality |  |  |  |
| Age |  | Number | Rate |
| 25 to 49 | Male | 270248.64  (-40483.14 to 381826.47) | 13.51  (-2.02 to 19.09) |
|  | Female | 104028.77  (-20334.73 to 151076.05) | 5.34  (-1.04 to 7.75) |
| 25 to 29 | Male | 12877.47  (-3487.72 to 18313.14) | 4.33  (-1.17 to 6.16) |
|  | Female | 6187.30  (-1522.06 to 8875.86) | 2.13  (-0.52 to 3.05) |
| 30 to 34 | Male | 27545.86  (-6023.06 to 38822.88) | 9.02  (-1.97 to 12.71) |
|  | Female | 10927.71  (-2646.90 to 15693.33) | 3.66  (-0.89 to 5.25) |
| 35 to 39 | Male | 44092.85  (-8460.41 to 62108.43) | 15.58  (-2.99 to 21.94) |
|  | Female | 16371.77  (-3680.81 to 23599.57) | 5.89  (-1.32 to 8.50) |
| 40 to 44 | Male | 75612.53  (-11460.55 to 106956.20) | 29.99  (-4.54 to 42.42) |
|  | Female | 27469.62  (-5649.49 to 39731.32) | 11.07  (-2.28 to 16.01) |
| 45 to 49 | Male | 110119.95  (-11381.46 to 156939.68) | 46.30  (-4.78 to 65.98) |
|  | Female | 43072.36  (-6835.47 to 63082.15) | 18.28  (-2.90 to 26.77) |

**Table S2**. Data on IHD related to Mortality Rate and DALYs Rate attributed to 13 dietary risks within 5 SDI regions in 1990 and 2021

| DALYs Rate | Dietary Risks | High SDI | High-middle SDI | Middle SDI | Low-middle SDI | Low SDI |
| --- | --- | --- | --- | --- | --- | --- |
|  | Diet low in whole grains | 94.56  (61.94 to 127.02) | 177.11  (114.25 to 236.34) | 164.50  (106.56 to 216.53) | 204.74  (132.07 to 269.65) | 135.15  (88.89 to 180.63) |
|  | Diet low in vegetables | 17.32  (8.92 to 26.49) | 17.52  (9.14 to 27.53) | 41.59  (21.94 to 61.81) | 63.46  (34.31 to 93.06) | 57.03  (29.81 to 83.58) |
|  | Diet low in seafood omega-3 fatty acids | 41.97  (9.70 to 69.81) | 54.81  (12.13 to 92.02) | 101.09  (23.68 to 161.62) | 165.75  (38.00 to 261.55) | 118.92  (27.89 to 187.87) |
|  | Diet low in polyunsaturated fatty acids | 36.52  (-124.94 to 133.65) | 94.54  (-363.55 to 348.00) | 117.07  (-465.50 to 421.39) | 148.13  (-596.42 to 524.91) | 83.46  (-327.63 to 301.30) |
|  | Diet low in nuts and seeds | 38.06  (12.26 to 61.71) | 87.97  (30.04 to 141.13) | 108.41  (34.63 to 166.89) | 173.32  (57.07 to 264.71) | 92.31  (30.51 to 140.42) |
|  | Diet low in legumes | 31.85  (-28.54 to 75.61) | 53.19  (-49.56 to 125.47) | 59.12  (-54.12 to 137.88) | 62.78  (-56.71 to 148.09) | 41.47  (-37.35 to 98.07) |
|  | Diet low in fruits | 41.23  (10.86 to 69.93) | 74.58  (19.70 to 121.42) | 123.80  (31.41 to 193.58) | 196.30  (46.83 to 304.60) | 108.75  (26.03 to 172.50) |
|  | Diet low in fiber | 35.46  (20.22 to 50.81) | 64.99  (36.43 to 95.01) | 100.06  (56.49 to 139.11) | 133.64  (74.64 to 186.43) | 67.36  (37.94 to 96.07) |
|  | Diet high in trans fatty acids | 1.78  (0.20 to 3.65) | 2.42  (0.27 to 5.19) | 17.08  (1.79 to 33.91) | 41.16  (3.95 to 78.04) | 10.02  (0.97 to 20.03) |
|  | Diet high in sugar-sweetened beverages | 3.88  (-0.87 to 8.74) | 1.94  (-0.42 to 4.35) | 2.27  (-0.54 to 4.98) | 1.59  (-0.38 to 3.61) | 0.52  (-0.13 to 1.18) |
|  | Diet high in sodium | 16.64  (2.77 to 45.52) | 57.67  (17.96 to 121.29) | 49.56  (11.91 to 117.36) | 39.77  (4.01 to 114.76) | 13.37  (0.50 to 44.93) |
|  | Diet high in red meat | 31.95  (-1.90 to 63.27) | 55.49  (-3.17 to 115.00) | 32.91  (-1.83 to 67.77) | 8.52  (-0.39 to 17.84) | 2.73  (-0.08 to 6.22) |
|  | Diet high in processed meat | 38.31  (16.91 to 57.94) | 17.30  (7.15 to 28.50) | 2.48  (0.95 to 4.09) | 4.82  (1.94 to 8.08) | 3.17  (1.22 to 5.24) |
| Mortality Rate |  |  |  |  |  |  |
|  | Diet low in whole grains | 1.94  (1.26 to 2.60) | 3.63  (2.34 to 4.86) | 3.33  (2.15 to 4.38) | 4.13  (2.65 to 5.44) | 2.73  (1.79 to 3.66) |
|  | Diet low in vegetables | 0.36  (0.18 to 0.55) | 0.36  (0.19 to 0.57) | 0.84  (0.44 to 1.25) | 1.29  (0.69 to 1.89) | 1.15  (0.60 to 1.70) |
|  | Diet low in seafood omega-3 fatty acids | 0.86  (0.20 to 1.43) | 1.12  (0.25 to 1.90) | 2.05  (0.48 to 3.29) | 3.35  (0.76 to 5.29) | 2.40  (0.56 to 3.80) |
|  | Diet low in polyunsaturated fatty acids | 0.75  (-2.55 to 2.73) | 1.94  (-7.43 to 7.12) | 2.38  (-9.39 to 8.58) | 3.00  (-12.00 to 10.62) | 1.69  (-6.58 to 6.11) |
|  | Diet low in nuts and seeds | 0.78  (0.25 to 1.26) | 1.80  (0.62 to 2.90) | 2.19  (0.70 to 3.38) | 3.50(1.15 to 5.34) | 1.86  (0.61 to 2.84) |
|  | Diet low in legumes | 0.65  (-0.59 to 1.55) | 1.10  (-1.02 to 2.59) | 1.20  (-1.09 to 2.80) | 1.27  (-1.14 to 3.00) | 0.84  (-0.75 to 1.99) |
|  | Diet low in fruits | 0.84  (0.22 to 1.42) | 1.52  (0.40 to 2.49) | 2.50  (0.63 to 3.91) | 3.97  (0.94 to 6.17) | 2.19  (0.52 to 3.48) |
|  | Diet low in fiber | 0.72  (0.41 to 1.04) | 1.32  (0.74 to 1.94) | 2.01  (1.13 to 2.80) | 2.69  (1.49 to 3.75) | 1.36  (0.75 to 1.94) |
|  | Diet high in trans fatty acids | 0.04  (0.00 to 0.07) | 0.05  (0.01 to 0.11) | 0.35  (0.04 to 0.70) | 0.83  (0.08 to 1.59) | 0.20  (0.02 to 0.41) |
|  | Diet high in sugar-sweetened beverages | 0.08  (-0.02 to 0.17) | 0.04  (-0.01 to 0.09) | 0.04  (-0.01 to 0.10) | 0.03  (-0.01 to 0.07) | 0.01  (-0.00 to 0.02) |
|  | Diet high in sodium | 0.35  (0.06 to 0.95) | 1.22  (0.38 to 2.54) | 1.04  (0.25 to 2.46) | 0.83  (0.09 to 2.36) | 0.28  (0.01 to 0.93) |
|  | Diet high in red meat | 0.65  (-0.04 to 1.30) | 1.13  (-0.06 to 2.36) | 0.66  (-0.04 to 1.36) | 0.17  (-0.01 to 0.36) | 0.05  (-0.00 to 0.13) |
|  | Diet high in processed meat | 0.79  (0.35 to 1.20) | 0.36  (0.15 to 0.59) | 0.05  (0.02 to 0.08) | 0.10  (0.04 to 0.16) | 0.06  (0.02 to 0.10) |

**Table S3**. Data on IHD related to Mortality Rate and DALYs Rate attributed to dietary risks by 204 countries and territories in 1990 and 2021

|  | **DALYs Rate** | | | **Mortality Rate** | | |
| --- | --- | --- | --- | --- | --- | --- |
| **location** | **1990** | **2021** | **EAPC** | **1990** | **2021** | **EAPC** |
| Afghanistan | 1712.42  (-131.32 to 2676.51) | 1199.94  (-65.62 to 1942.65) | -0.41 (-0.82 to 0) | 36.67  (-2.83 to 57.76) | 6.94  (-0.64 to 10.79) | -0.47 (-0.99 to 0.05) |
| Albania | 337.97  (70.59 to 441.03) | 320.25  (33.45 to 452.97) | -0.06 (-0.4 to 0.28) | 6.70  (1.44 to 8.77) | 2.45  (1.07 to 3.48) | 0.12 (-0.23 to 0.47) |
| Algeria | 792.11  (43.56 to 1148.22) | 556.68  (39.90 to 833.96) | -1.67 (-1.95 to -1.4) | 15.76  (0.86 to 22.83) | 6.04  (-4.66 to 11.27) | -1.61 (-1.88 to -1.35) |
| American Samoa | 649.34  (-226.42 to 1087.12) | 1007.36  (-445.19 to 1730.83) | 1.72 (1.61 to 1.82) | 13.31  (-4.62 to 22.20) | 10.99  (-9.34 to 21.33) | 1.82 (1.7 to 1.93) |
| Andorra | 291.41  (-15.93 to 421.35) | 120.76  (2.52 to 207.25) | -1.58 (-1.87 to -1.28) | 5.70  (-0.29 to 8.27) | 15.19  (2.33 to 24.17) | -1.53 (-1.85 to -1.21) |
| Angola | 211.00  (6.81 to 343.55) | 222.26  (-37.64 to 382.54) | -1.01 (-1.13 to -0.89) | 4.31  (0.12 to 7.06) | 11.83  (-8.81 to 21.22) | -1.06 (-1.18 to -0.93) |
| Antigua and Barbuda | 287.34  (28.23 to 446.81) | 102.43  (24.83 to 149.82) | -2.51 (-2.78 to -2.25) | 5.99  (0.59 to 9.33) | 5.55  (-2.38 to 9.12) | -2.5 (-2.79 to -2.2) |
| Argentina | 288.35  (109.06 to 383.28) | 194.57  (-3.50 to 260.80) | -3.39 (-3.7 to -3.08) | 5.78  (2.20 to 7.69) | 13.09  (-11.60 to 22.82) | -3.44 (-3.74 to -3.14) |
| Armenia | 561.82  (-0.73 to 720.93) | 634.97  (-71.46 to 863.50) | -1.76 (-2.15 to -1.37) | 11.68  (0.02 to 15.02) | 9.01  (-3.79 to 14.88) | -1.63 (-2.05 to -1.21) |
| Australia | 758.33  (-45.29 to 979.18) | 134.03  (-44.88 to 200.05) | -2.94 (-3.13 to -2.75) | 15.31  (-0.79 to 19.78) | 4.82  (-3.53 to 9.68) | -2.95 (-3.14 to -2.77) |
| Austria | 323.58  (-111.86 to 463.06) | 104.77  (-45.80 to 159.51) | -4.1 (-4.41 to -3.8) | 6.73  (-2.31 to 9.67) | 7.98  (-4.68 to 14.79) | -4.16 (-4.47 to -3.85) |
| Azerbaijan | 363.66  (-197.69 to 538.97) | 656.02  (-158.69 to 970.46) | -2.32 (-2.73 to -1.91) | 7.65  (-4.15 to 11.37) | 3.96  (-2.92 to 7.56) | -2.15 (-2.59 to -1.71) |
| Bahamas | 1008.28  (-135.28 to 1343.78) | 310.90  (14.74 to 481.42) | -1.47 (-1.57 to -1.37) | 20.24  (-2.56 to 26.95) | 7.04  (-3.85 to 12.95) | -1.36 (-1.47 to -1.25) |
| Bahrain | 479.96  (116.32 to 643.19) | 373.83  (-30.01 to 581.56) | -2.95 (-3.42 to -2.48) | 9.75  (2.34 to 13.12) | 28.19  (2.00 to 44.85) | -2.99 (-3.47 to -2.5) |
| Bangladesh | 693.02  (-77.19 to 1008.06) | 475.02  (-152.39 to 784.80) | -0.67 (-0.86 to -0.48) | 13.58  (-1.52 to 19.84) | 9.07  (-2.02 to 15.74) | -0.63 (-0.81 to -0.45) |
| Barbados | 619.44  (-108.67 to 895.86) | 139.08  (-13.62 to 224.76) | -2.09 (-2.34 to -1.83) | 12.59  (-2.18 to 18.37) | 37.26  (-14.46 to 63.46) | -2.04 (-2.3 to -1.78) |
| Belarus | 269.44  (-4.05 to 389.65) | 1153.46  (-188.05 to 1663.37) | -1.23 (-1.89 to -0.56) | 5.47  (-0.07 to 7.95) | 25.32  (-11.84 to 44.29) | -1.14 (-1.82 to -0.45) |
| Belgium | 1136.07  (-138.63 to 1488.30) | 101.00  (13.44 to 142.10) | -4.42 (-4.66 to -4.19) | 23.60  (-2.85 to 30.87) | 15.68  (-5.50 to 26.85) | -4.49 (-4.75 to -4.23) |
| Belize | 361.51  (16.51 to 498.22) | 139.98  (-4.96 to 223.12) | -1.85 (-2.19 to -1.52) | 7.47  (0.32 to 10.36) | 34.73  (-12.73 to 61.34) | -1.85 (-2.2 to -1.51) |
| Benin | 210.36  (-52.20 to 333.14) | 120.20  (-57.78 to 205.14) | -0.2 (-0.5 to 0.1) | 4.22  (-1.04 to 6.69) | 22.12  (-4.59 to 37.51) | -0.24 (-0.55 to 0.07) |
| Bermuda | 125.52  (-50.08 to 203.74) | 200.45  (-21.69 to 322.30) | -2.83 (-3.13 to -2.52) | 2.56  (-1.01 to 4.17) | 4.15  (-0.46 to 6.70) | -2.81 (-3.13 to -2.5) |
| Bhutan | 475.13  (-50.87 to 722.67) | 390.64  (-97.41 to 656.84) | -0.69 (-0.84 to -0.55) | 9.85  (-1.03 to 15.06) | 10.79  (-4.60 to 19.74) | -0.71 (-0.86 to -0.56) |
| Bolivia (Plurinational State of) | 415.39  (-79.79 to 687.07) | 198.80  (-7.66 to 338.41) | -2.68 (-3.17 to -2.18) | 8.30  (-1.60 to 13.75) | 52.94  (-22.09 to 93.00) | -2.7 (-3.19 to -2.2) |
| Bosnia and Herzegovina | 397.13  (7.74 to 658.24) | 315.47  (-34.27 to 503.27) | -2.89 (-3.24 to -2.53) | 7.88  (0.15 to 13.18) | 20.19  (-7.28 to 33.55) | -2.86 (-3.23 to -2.49) |
| Botswana | 603.09  (17.90 to 811.93) | 228.60  (-21.36 to 390.18) | -0.8 (-1.15 to -0.46) | 12.38  (0.48 to 16.69) | 15.36  (-5.96 to 25.56) | -0.88 (-1.24 to -0.53) |
| Brazil | 245.78  (1.02 to 411.38) | 283.60  (76.33 to 406.31) | -1.68 (-1.81 to -1.56) | 5.08  (0.02 to 8.57) | 3.44  (0.83 to 5.63) | -1.69 (-1.8 to -1.58) |
| Brunei Darussalam | 449.57  (166.58 to 606.01) | 435.27  (-173.37 to 695.17) | -0.47 (-0.94 to 0.01) | 9.15  (3.38 to 12.35) | 4.70  (0.53 to 7.27) | -0.26 (-0.72 to 0.2) |
| Bulgaria | 470.25  (-240.47 to 765.63) | 1023.34  (231.62 to 1454.21) | -1.49 (-1.81 to -1.17) | 9.16  (-4.65 to 14.94) | 23.79  (-9.39 to 43.92) | -1.49 (-1.83 to -1.15) |
| Burkina Faso | 1250.79  (136.43 to 1642.93) | 137.96  (-27.98 to 227.33) | -0.19 (-0.35 to -0.03) | 26.10  (2.98 to 34.28) | 3.88  (0.36 to 6.03) | -0.25 (-0.41 to -0.09) |
| Burundi | 148.49  (-18.71 to 240.22) | 266.65  (-7.20 to 420.51) | -1.62 (-1.97 to -1.27) | 3.06  (-0.38 to 4.98) | 1.20  (0.07 to 2.19) | -1.72 (-2.08 to -1.37) |
| Cabo Verde | 335.60  (-3.32 to 549.81) | 186.81  (-28.23 to 326.20) | 0.3 (-0.06 to 0.67) | 6.76  (-0.04 to 11.24) | 38.05  (-15.33 to 64.66) | 0.46 (0.09 to 0.84) |
| Cambodia | 163.86  (-48.60 to 264.77) | 299.61  (-232.41 to 555.68) | -1.44 (-1.58 to -1.31) | 3.20  (-0.94 to 5.18) | 3.88  (0.20 to 6.04) | -1.41 (-1.53 to -1.28) |
| Cameroon | 417.88  (-212.95 to 648.15) | 152.45  (-125.50 to 293.94) | 0.9 (0.28 to 1.53) | 8.40  (-4.22 to 13.14) | 20.91  (-9.13 to 37.57) | 0.85 (0.22 to 1.49) |
| Canada | 115.18  (-79.02 to 215.18) | 127.02  (-52.42 to 201.69) | -3.06 (-3.31 to -2.81) | 2.36  (-1.63 to 4.43) | 32.16  (-10.63 to 53.23) | -3.09 (-3.36 to -2.81) |
| Central African Republic | 308.24  (-100.42 to 457.18) | 421.04  (-94.75 to 796.62) | 0.18 (0.07 to 0.29) | 6.39  (-2.05 to 9.50) | 7.69  (1.40 to 12.74) | 0.19 (0.09 to 0.3) |
| Chad | 386.80  (-108.55 to 732.39) | 202.83  (-0.01 to 324.73) | 0.39 (0.1 to 0.68) | 8.14  (-2.28 to 15.50) | 17.59  (-0.75 to 28.99) | 0.36 (0.06 to 0.66) |
| Chile | 176.00  (4.22 to 263.85) | 165.05  (10.25 to 221.96) | -0.34 (-0.51 to -0.16) | 3.60  (0.09 to 5.45) | 4.63  (-1.61 to 8.71) | -0.3 (-0.49 to -0.11) |
| China | 203.06  (24.05 to 262.72) | 342.94  (-34.82 to 529.21) | 0.42 (0.25 to 0.59) | 4.13  (0.50 to 5.36) | 12.10  (-4.63 to 21.14) | 0.53 (0.34 to 0.71) |
| Colombia | 307.95  (-23.40 to 430.98) | 189.25  (-5.61 to 285.40) | -1.94 (-2.16 to -1.71) | 6.11  (-0.41 to 8.57) | 13.17  (-1.41 to 17.90) | -1.98 (-2.19 to -1.77) |
| Comoros | 352.62  (-68.88 to 496.99) | 223.24  (-42.98 to 373.43) | 0.05 (-0.3 to 0.4) | 7.08  (-1.35 to 9.99) | 29.43  (-8.18 to 49.00) | 0.04 (-0.32 to 0.4) |
| Congo | 197.87  (-40.22 to 341.37) | 358.86  (-145.99 to 678.39) | -0.39 (-0.64 to -0.15) | 3.97  (-0.78 to 6.94) | 14.46  (-1.01 to 19.58) | -0.39 (-0.65 to -0.14) |
| Cook Islands | 368.70  (-140.99 to 673.16) | 517.00  (-221.26 to 940.92) | 0.14 (-0.17 to 0.45) | 7.69  (-2.94 to 14.07) | 35.87  (-15.36 to 60.15) | 0.22 (-0.1 to 0.53) |
| Costa Rica | 638.17  (-253.84 to 1097.09) | 235.99  (31.72 to 334.03) | -0.87 (-1.24 to -0.5) | 13.24  (-5.20 to 22.72) | 11.36  (-0.90 to 15.92) | -0.88 (-1.23 to -0.53) |
| Coted'Ivoire | 254.61  (1.59 to 347.78) | 238.48  (-174.46 to 436.88) | 0.5 (0.28 to 0.72) | 5.05  (0.03 to 6.92) | 13.43  (-3.21 to 19.89) | 0.46 (0.24 to 0.68) |
| Croatia | 199.71  (-128.75 to 348.37) | 257.65  (30.89 to 355.49) | -3.25 (-3.49 to -3.02) | 4.06  (-2.61 to 7.16) | 10.14  (-0.79 to 14.81) | -3.25 (-3.51 to -2.99) |
| Cuba | 646.97  (34.64 to 855.51) | 251.64  (-41.01 to 401.43) | -2.27 (-2.53 to -2) | 13.35  (0.79 to 17.65) | 17.54  (-2.95 to 24.79) | -2.18 (-2.46 to -1.91) |
| Cyprus | 475.16  (-94.91 to 680.15) | 181.83  (20.98 to 265.50) | -2.41 (-2.72 to -2.11) | 9.75  (-1.93 to 14.00) | 8.65  (0.10 to 12.25) | -2.41 (-2.71 to -2.11) |
| Czechia | 312.70  (93.99 to 435.43) | 258.90  (-60.33 to 376.10) | -4.35 (-4.73 to -3.96) | 6.52  (1.95 to 9.11) | 15.34  (0.07 to 20.94) | -4.5 (-4.89 to -4.11) |
| Democratic People's Republic of Korea | 948.94  (-219.11 to 1287.18) | 743.18  (111.57 to 1185.82) | 1.42 (1.25 to 1.58) | 20.10  (-4.47 to 27.29) | 6.53  (0.68 to 9.33) | 1.52 (1.36 to 1.68) |
| Democratic Republic of the Congo | 475.73  (81.44 to 769.30) | 216.84  (-67.21 to 376.45) | 0.11 (0.02 to 0.21) | 9.64  (1.73 to 15.63) | 19.21  (-3.27 to 29.90) | 0.13 (0.03 to 0.23) |
| Denmark | 199.61  (-112.33 to 372.33) | 104.73  (-22.13 to 149.42) | -4.72 (-4.87 to -4.56) | 4.12  (-2.31 to 7.72) | 21.62  (4.97 to 30.79) | -4.79 (-4.94 to -4.63) |
| Djibouti | 442.76  (-77.52 to 616.85) | 338.43  (-14.54 to 560.85) | 1.36 (1.12 to 1.59) | 9.32  (-1.63 to 13.01) | 5.33  (-1.19 to 7.77) | 1.36 (1.13 to 1.59) |
| Dominica | 216.91  (10.72 to 357.32) | 178.56  (-34.09 to 290.76) | -0.3 (-0.47 to -0.13) | 4.36  (0.23 to 7.31) | 6.53  (-0.65 to 10.48) | -0.29 (-0.47 to -0.1) |
| Dominican Republic | 198.99  (-41.20 to 296.60) | 472.10  (156.63 to 729.67) | 0.97 (0.71 to 1.23) | 4.13  (-0.83 to 6.20) | 5.34  (0.69 to 7.37) | 1.08 (0.83 to 1.33) |
| Ecuador | 413.03  (98.52 to 592.47) | 264.20  (10.95 to 390.78) | -0.87 (-1.31 to -0.43) | 8.13  (1.94 to 11.66) | 7.60  (0.51 to 11.71) | -0.85 (-1.3 to -0.39) |
| Egypt | 307.92  (16.61 to 413.90) | 969.37  (-371.89 to 1598.89) | -0.85 (-1.03 to -0.67) | 6.02  (0.34 to 8.12) | 5.24  (-0.73 to 7.35) | -0.86 (-1.01 to -0.71) |
| El Salvador | 1309.73  (-440.79 to 1859.36) | 319.86  (-85.37 to 529.62) | -0.65 (-1.1 to -0.2) | 25.78  (-8.69 to 36.72) | 8.98  (1.74 to 12.58) | -0.59 (-1.05 to -0.13) |
| Equatorial Guinea | 401.61  (-117.52 to 585.11) | 167.31  (-137.81 to 362.90) | -3.1 (-3.76 to -2.44) | 7.93  (-2.28 to 11.60) | 9.52  (0.04 to 14.08) | -3.25 (-3.94 to -2.56) |
| Eritrea | 361.60  (-109.88 to 639.75) | 345.13  (-76.91 to 611.53) | 0.39 (0.31 to 0.47) | 7.64  (-2.33 to 13.54) | 7.11  (0.31 to 10.42) | 0.32 (0.23 to 0.41) |
| Estonia | 312.19  (-37.24 to 518.61) | 175.38  (-29.00 to 255.94) | -6.89 (-7.42 to -6.36) | 6.45  (-0.76 to 10.79) | 11.79  (-1.00 to 16.61) | -6.97 (-7.52 to -6.42) |
| Eswatini | 1112.30  (-69.97 to 1501.57) | 345.20  (-134.83 to 648.50) | 2.46 (1.67 to 3.25) | 23.43  (-1.45 to 31.57) | 2.39  (0.17 to 3.45) | 2.43 (1.63 to 3.24) |
| Ethiopia | 188.91  (-48.75 to 320.85) | 166.38  (1.64 to 244.44) | -2.86 (-3.14 to -2.57) | 3.94  (-1.01 to 6.81) | 7.82  (0.56 to 11.30) | -2.98 (-3.27 to -2.68) |
| Fiji | 343.14  (37.99 to 543.91) | 1354.67  (94.54 to 2147.36) | -0.46 (-0.58 to -0.35) | 6.95  (0.78 to 11.08) | 24.42  (-3.93 to 35.39) | -0.44 (-0.55 to -0.33) |
| Finland | 1603.83  (408.15 to 2302.90) | 138.38  (-50.91 to 197.65) | -5.14 (-5.29 to -4.98) | 33.33  (8.45 to 48.05) | 10.75  (1.06 to 14.66) | -5.25 (-5.42 to -5.08) |
| France | 650.15  (-218.14 to 907.74) | 104.20  (8.75 to 141.75) | -2.55 (-2.84 to -2.26) | 13.57  (-4.50 to 19.01) | 3.60  (-0.58 to 5.30) | -2.62 (-2.94 to -2.3) |
| Gabon | 221.35  (-25.61 to 305.71) | 188.40  (-178.25 to 403.23) | -0.29 (-0.55 to -0.04) | 4.49  (-0.53 to 6.27) | 11.21  (0.15 to 15.39) | -0.27 (-0.53 to 0) |
| Gambia | 191.03  (-153.31 to 381.33) | 241.06  (-101.69 to 413.75) | 0.66 (0.4 to 0.92) | 3.96  (-3.16 to 7.91) | 16.02  (-3.66 to 22.78) | 0.65 (0.39 to 0.91) |
| Georgia | 185.57  (-77.34 to 320.54) | 692.72  (-50.84 to 935.89) | -3.66 (-4.52 to -2.8) | 3.77  (-1.58 to 6.57) | 17.51  (-1.23 to 23.99) | -3.55 (-4.41 to -2.68) |
| Germany | 1243.18  (-215.84 to 1644.64) | 139.46  (-14.04 to 192.44) | -3.62 (-3.91 to -3.33) | 25.60  (-4.25 to 33.92) | 1.53  (0.19 to 2.38) | -3.59 (-3.91 to -3.27) |
| Ghana | 477.43  (-112.59 to 668.12) | 174.32  (-110.51 to 328.64) | -2.63 (-3.04 to -2.22) | 9.97  (-2.35 to 14.02) | 8.87  (-3.52 to 14.25) | -2.66 (-3.07 to -2.25) |
| Greece | 322.31  (-194.44 to 531.04) | 369.26  (-34.36 to 531.03) | -0.23 (-0.54 to 0.08) | 6.62  (-4.00 to 11.04) | 20.97  (-2.78 to 34.11) | -0.15 (-0.44 to 0.15) |
| Greenland | 415.87  (-81.14 to 597.16) | 222.33  (25.05 to 342.89) | -3.26 (-3.58 to -2.93) | 8.54  (-1.64 to 12.29) | 2.58  (0.01 to 3.66) | -3.17 (-3.52 to -2.82) |
| Grenada | 640.51  (-15.44 to 962.13) | 222.91  (42.03 to 332.23) | -2.14 (-2.44 to -1.84) | 13.57  (-0.33 to 20.50) | 2.49  (0.06 to 4.30) | -2.01 (-2.3 to -1.71) |
| Guam | 410.25  (14.29 to 579.83) | 961.20  (-349.79 to 1595.11) | 2.71 (2.46 to 2.96) | 8.16  (0.29 to 11.59) | 3.00  (-0.82 to 4.48) | 2.85 (2.58 to 3.12) |
| Guatemala | 453.25  (-162.68 to 762.76) | 261.92  (-5.43 to 394.54) | -2.25 (-2.92 to -1.57) | 9.21  (-3.32 to 15.49) | 3.36  (-0.93 to 5.33) | -2.25 (-2.92 to -1.57) |
| Guinea | 461.93  (-92.56 to 643.71) | 173.48  (-116.36 to 311.50) | 0.58 (0.46 to 0.69) | 9.00  (-1.78 to 12.54) | 2.77  (-0.91 to 4.15) | 0.52 (0.4 to 0.64) |
| Guinea-Bissau | 158.88  (-87.71 to 281.15) | 373.06  (-137.75 to 643.21) | 0.23 (0.18 to 0.28) | 3.26  (-1.79 to 5.79) | 2.12  (-0.90 to 3.25) | 0.17 (0.12 to 0.23) |
| Guyana | 352.00  (-146.84 to 601.38) | 359.73  (-182.67 to 641.91) | -1.41 (-1.78 to -1.03) | 7.29  (-3.01 to 12.44) | 7.82  (-0.69 to 11.26) | -1.31 (-1.71 to -0.92) |
| Haiti | 598.06  (-275.42 to 928.66) | 617.00  (80.75 to 998.99) | -0.3 (-0.5 to -0.1) | 12.27  (-5.63 to 19.11) | 2.11  (0.17 to 2.88) | -0.32 (-0.52 to -0.13) |
| Honduras | 705.63  (-39.76 to 1079.84) | 248.20  (-64.16 to 436.39) | -1.13 (-1.37 to -0.89) | 14.47  (-0.79 to 22.20) | 2.06  (0.27 to 2.92) | -1.02 (-1.25 to -0.79) |
| Hungary | 311.81  (-76.31 to 477.68) | 424.42  (81.13 to 592.00) | -4.72 (-5.01 to -4.44) | 6.27  (-1.51 to 9.64) | 2.15  (-0.45 to 3.11) | -4.71 (-5 to -4.41) |
| Iceland | 1360.98  (95.46 to 1759.27) | 165.88  (-14.73 to 241.86) | -3.18 (-3.44 to -2.93) | 28.36  (2.23 to 36.66) | 2.80  (-1.00 to 4.02) | -3.26 (-3.52 to -3) |
| India | 360.87  (-0.06 to 481.79) | 767.40  (-129.37 to 1073.50) | -0.03 (-0.15 to 0.09) | 7.33  (0.03 to 9.78) | 3.78  (0.45 to 5.58) | 0.02 (-0.09 to 0.14) |
| Indonesia | 794.39  (-153.94 to 1091.03) | 578.49  (-441.61 to 1035.01) | 0.7 (0.53 to 0.86) | 16.05  (-3.04 to 22.11) | 2.88  (-0.29 to 3.99) | 0.8 (0.64 to 0.97) |
| Iran (Islamic Republic of) | 510.09  (-224.97 to 788.47) | 453.90  (38.17 to 627.71) | -1.17 (-1.32 to -1.02) | 10.16  (-4.37 to 15.67) | 2.72  (-1.03 to 4.23) | -1.21 (-1.36 to -1.05) |
| Iraq | 622.80  (94.99 to 822.93) | 561.85  (37.43 to 869.42) | -0.82 (-1.01 to -0.63) | 12.51  (1.89 to 16.51) | 2.64  (0.62 to 3.53) | -0.81 (-0.98 to -0.63) |
| Ireland | 686.34  (-27.61 to 1000.65) | 131.40  (-50.29 to 201.59) | -3.95 (-4.09 to -3.8) | 14.24  (-0.58 to 20.78) | 4.87  (1.18 to 6.61) | -4.01 (-4.17 to -3.85) |
| Israel | 446.92  (-130.47 to 621.62) | 53.59  (15.22 to 76.11) | -5.42 (-5.66 to -5.19) | 9.38  (-2.73 to 13.07) | 3.37  (-0.29 to 4.93) | -5.59 (-5.84 to -5.34) |
| Italy | 244.59  (46.91 to 348.08) | 129.31  (30.36 to 171.58) | -2.38 (-2.54 to -2.23) | 4.96  (0.96 to 7.07) | 1.58  (0.03 to 2.27) | -2.4 (-2.56 to -2.25) |
| Jamaica | 286.62  (31.95 to 384.60) | 117.58  (-17.14 to 198.84) | 0.12 (-0.4 to 0.65) | 5.90  (0.67 to 7.94) | 2.04  (-0.31 to 2.92) | 0.26 (-0.29 to 0.81) |
| Japan | 115.02  (-33.39 to 176.58) | 124.78  (0.71 to 175.96) | -0.59 (-0.81 to -0.37) | 2.28  (-0.65 to 3.49) | 1.04  (0.30 to 1.48) | -0.55 (-0.75 to -0.35) |
| Jordan | 161.14  (-0.85 to 233.40) | 321.80  (55.89 to 467.90) | -2.01 (-2.18 to -1.84) | 3.28  (-0.01 to 4.75) | 1.75  (0.59 to 2.28) | -2.01 (-2.18 to -1.85) |
| Kazakhstan | 536.89  (-19.27 to 774.35) | 417.32  (4.01 to 589.55) | -4.97 (-6.13 to -3.8) | 10.84  (-0.40 to 15.75) | 3.23  (-0.50 to 4.83) | -4.92 (-6.1 to -3.73) |
| Kenya | 1107.59  (47.28 to 1427.86) | 138.43  (-43.37 to 226.09) | 2.12 (1.61 to 2.64) | 22.59  (1.13 to 29.11) | 1.33  (-0.16 to 1.92) | 2.21 (1.69 to 2.74) |
| Kiribati | 78.75  (-23.87 to 123.67) | 1245.70  (-580.45 to 2169.18) | 0.52 (0.4 to 0.64) | 1.51  (-0.46 to 2.39) | 2.83  (-0.07 to 4.16) | 0.52 (0.39 to 0.64) |
| Kuwait | 1005.58  (-439.67 to 1644.15) | 558.98  (60.57 to 838.42) | -0.7 (-1.17 to -0.23) | 20.59  (-9.00 to 33.93) | 3.70  (0.13 to 4.89) | -0.68 (-1.17 to -0.18) |
| Kyrgyzstan | 664.54  (61.29 to 922.68) | 557.63  (-45.40 to 778.88) | -2.73 (-3.36 to -2.09) | 13.21  (1.24 to 18.32) | 1.48  (-0.34 to 2.13) | -2.59 (-3.24 to -1.94) |
| Lao People's Democratic Republic | 842.09  (-45.13 to 1116.59) | 550.74  (-471.34 to 1067.35) | -2.66 (-2.85 to -2.47) | 16.81  (-0.83 to 22.32) | 3.99  (-0.06 to 5.42) | -2.7 (-2.89 to -2.51) |
| Latvia | 1122.39  (-342.41 to 1795.68) | 506.91  (51.24 to 688.43) | -4.64 (-5.21 to -4.06) | 22.69  (-6.69 to 36.43) | 3.35  (0.21 to 4.51) | -4.63 (-5.21 to -4.05) |
| Lebanon | 1411.25  (111.17 to 1764.74) | 272.78  (74.52 to 404.97) | -3.17 (-3.79 to -2.55) | 29.85  (2.41 to 37.34) | 4.85  (1.02 to 6.39) | -3.36 (-3.99 to -2.73) |
| Lesotho | 743.52  (300.66 to 1098.18) | 260.27  (-61.45 to 526.81) | 3.9 (3.2 to 4.61) | 15.31  (6.21 to 22.81) | 2.60  (-1.06 to 4.13) | 3.89 (3.16 to 4.63) |
| Liberia | 95.06  (-17.97 to 155.23) | 264.87  (-82.60 to 445.28) | 0.88 (0.65 to 1.12) | 1.96  (-0.37 to 3.24) | 5.69  (2.32 to 7.39) | 0.82 (0.57 to 1.07) |
| Libya | 170.74  (-91.68 to 283.28) | 902.24  (231.60 to 1365.24) | 2.5 (2.28 to 2.72) | 3.46  (-1.84 to 5.77) | 2.04  (0.50 to 3.00) | 2.64 (2.41 to 2.87) |
| Lithuania | 516.33  (91.99 to 759.56) | 524.15  (6.85 to 718.35) | -2.91 (-3.43 to -2.38) | 10.06  (1.81 to 14.88) | 2.80  (-0.27 to 4.62) | -2.84 (-3.37 to -2.3) |
| Luxembourg | 1257.55  (85.41 to 1626.18) | 100.54  (-15.90 to 142.49) | -5.18 (-5.46 to -4.91) | 26.40  (1.87 to 34.17) | 5.23  (-0.85 to 8.33) | -5.22 (-5.5 to -4.94) |
| Madagascar | 421.63  (-44.95 to 570.04) | 379.98  (-105.60 to 629.91) | 0.46 (0.29 to 0.63) | 8.79  (-0.92 to 11.88) | 7.46  (-3.77 to 13.37) | 0.47 (0.3 to 0.65) |
| Malawi | 305.07  (-101.58 to 460.98) | 229.10  (-109.80 to 388.49) | 0.5 (0.18 to 0.82) | 6.08  (-1.99 to 9.28) | 2.35  (-0.34 to 4.04) | 0.49 (0.17 to 0.81) |
| Malaysia | 175.61  (-78.04 to 285.81) | 439.19  (-188.49 to 723.57) | 0.5 (0.28 to 0.72) | 3.48  (-1.54 to 5.72) | 4.59  (0.86 to 6.85) | 0.57 (0.36 to 0.78) |
| Maldives | 396.87  (-164.23 to 615.29) | 288.56  (-126.36 to 470.64) | -2.94 (-3.37 to -2.51) | 8.02  (-3.19 to 12.42) | 5.64  (-1.49 to 8.88) | -3.09 (-3.51 to -2.67) |
| Mali | 626.33  (-147.09 to 1047.88) | 128.13  (-45.86 to 220.42) | -0.89 (-1.03 to -0.75) | 12.54  (-2.85 to 20.99) | 2.89  (-0.60 to 4.62) | -0.97 (-1.11 to -0.83) |
| Malta | 171.67  (-35.47 to 278.60) | 232.77  (56.57 to 314.36) | -2.58 (-2.82 to -2.33) | 3.50  (-0.73 to 5.70) | 12.61  (1.64 to 20.70) | -2.62 (-2.89 to -2.35) |
| Marshall Islands | 413.85  (150.61 to 526.21) | 1807.33  (-700.85 to 3060.27) | 1.5 (1.32 to 1.68) | 8.52  (3.10 to 10.81) | 9.91  (3.69 to 15.14) | 1.51 (1.32 to 1.71) |
| Mauritania | 1093.93  (-374.59 to 1670.48) | 192.97  (28.85 to 318.67) | -1.52 (-1.65 to -1.4) | 22.22  (-7.69 to 33.92) | 6.39  (0.30 to 9.96) | -1.56 (-1.69 to -1.44) |
| Mauritius | 280.32  (44.72 to 415.71) | 582.93  (256.74 to 778.42) | -1.04 (-1.3 to -0.77) | 5.75  (0.90 to 8.58) | 7.05(  1.77 to 11.18) | -1.11 (-1.38 to -0.83) |
| Mexico | 896.02  (416.59 to 1168.71) | 369.33  (-87.74 to 574.12) | 1.72 (1.34 to 2.09) | 18.12  (8.51 to 23.65) | 9.57  (3.19 to 14.85) | 1.74 (1.37 to 2.12) |
| Micronesia (Federated States of) | 221.25  (-42.83 to 319.72) | 1676.22  (-614.57 to 2953.96) | 0.66 (0.61 to 0.71) | 4.38  (-0.83 to 6.34) | 2.82  (-0.09 to 4.51) | 0.7 (0.64 to 0.76) |
| Monaco | 1397.47  (-408.80 to 2292.30) | 165.74  (40.40 to 269.29) | -2.12 (-2.3 to -1.94) | 28.49  (-8.46 to 46.85) | 3.67  (-0.67 to 5.98) | -2.13 (-2.32 to -1.94) |
| Mongolia | 329.15  (69.35 to 515.11) | 735.23  (0.35 to 999.55) | -0.07 (-0.52 to 0.37) | 6.92  (1.46 to 10.93) | 3.11  (-1.13 to 5.24) | 0.04 (-0.41 to 0.48) |
| Montenegro | 673.47  (42.06 to 943.37) | 456.10  (-2.07 to 675.75) | -1.38 (-1.86 to -0.9) | 13.84  (0.92 to 19.41) | 3.93  (-0.14 to 6.75) | -1.29 (-1.8 to -0.79) |
| Morocco | 600.61  (7.86 to 818.27) | 734.83  (65.09 to 1226.21) | -1.49 (-1.61 to -1.37) | 12.21  (0.26 to 16.83) | 6.43  (-1.68 to 10.69) | -1.38 (-1.5 to -1.27) |
| Mozambique | 1068.02  (91.98 to 1514.00) | 142.20  (-7.94 to 231.34) | 2.32 (2 to 2.63) | 21.52  (1.84 to 30.49) | 5.14  (-0.10 to 7.78) | 2.37 (2.05 to 2.7) |
| Myanmar | 88.73  (2.98 to 131.21) | 392.89  (-235.65 to 728.58) | -2.49 (-2.72 to -2.27) | 1.76  (0.06 to 2.64) | 7.40  (-1.74 to 11.55) | -2.41 (-2.63 to -2.19) |
| Namibia | 790.85  (-326.38 to 1354.17) | 272.24  (-50.45 to 478.26) | 0.16 (-0.32 to 0.65) | 15.69  (-6.31 to 26.84) | 3.28  (0.53 to 5.00) | 0.16 (-0.32 to 0.64) |
| Nauru | 227.67  (12.86 to 336.80) | 2587.11  (-1074.20 to 4542.85) | 0.32 (-0.15 to 0.81) | 4.75  (0.27 to 7.04) | 5.13  (-1.29 to 9.06) | 0.3 (-0.19 to 0.79) |
| Nepal | 2303.08  (-837.82 to 3989.28) | 510.09  (-31.34 to 818.86) | -0.58 (-0.74 to -0.41) | 47.40  (-17.41 to 82.50) | 4.70  (0.63 to 6.73) | -0.55 (-0.73 to -0.37) |
| Netherlands | 648.93  (-104.22 to 994.17) | 78.43  (1.71 to 111.89) | -5.39 (-5.76 to -5.02) | 13.14  (-2.11 to 20.17) | 4.55  (0.45 to 6.62) | -5.47 (-5.85 to -5.09) |
| New Zealand | 367.84  (28.77 to 511.95) | 144.68  (-39.39 to 215.92) | -3.57 (-3.75 to -3.38) | 7.68  (0.60 to 10.71) | 13.19  (1.93 to 20.70) | -3.55 (-3.73 to -3.36) |
| Nicaragua | 407.34(  -126.82 to 594.07) | 231.18  (22.65 to 333.76) | 0.14 (-0.09 to 0.36) | 8.54  (-2.64 to 12.49) | 5.18  (0.22 to 7.76) | 0.14 (-0.08 to 0.36) |
| Niger | 228.86  (26.95 to 310.74) | 89.91  (-23.60 to 163.79) | -0.58 (-0.69 to -0.48) | 4.49  (0.53 to 6.11) | 3.78  (-0.10 to 5.72) | -0.62 (-0.73 to -0.51) |
| Nigeria | 116.06  (-27.52 to 206.82) | 153.33  (-86.89 to 260.87) | -0.41 (-0.59 to -0.23) | 2.37  (-0.56 to 4.26) | 9.08  (0.73 to 12.55) | -0.46 (-0.64 to -0.27) |
| Niue | 180.57  (-59.58 to 314.08) | 1136.55  (-458.93 to 2112.77) | -0.24 (-0.46 to -0.02) | 3.73  (-1.22 to 6.50) | 7.34  (-0.61 to 11.51) | -0.21 (-0.42 to 0.01) |
| North Macedonia | 1126.17  (-411.42 to 2045.39) | 364.96  (23.05 to 557.50) | -2.55 (-2.95 to -2.15) | 23.48  (-8.50 to 42.58) | 6.44  (1.09 to 9.41) | -2.55 (-2.97 to -2.13) |
| Northern Mariana Islands | 686.26  (40.71 to 938.37) | 727.24  (-282.44 to 1210.64) | 1.86 (1.43 to 2.28) | 14.23  (0.96 to 19.45) | 4.27  (0.97 to 6.60) | 2.07 (1.61 to 2.53) |
| Norway | 549.50  (-173.86 to 1008.36) | 86.11  (29.71 to 111.18) | -5.65 (-5.8 to -5.49) | 11.33  (-3.55 to 20.80) | 5.34  (1.47 to 7.95) | -5.76 (-5.92 to -5.59) |
| Oman | 490.08  (169.50 to 618.03) | 359.70  (-145.19 to 581.12) | -1.94 (-2.06 to -1.82) | 10.31  (3.54 to 13.03) | 15.22  (1.34 to 25.57) | -2.09 (-2.21 to -1.96) |
| Pakistan | 648.26  (-146.13 to 1012.97) | 948.79  (16.85 to 1406.46) | 1.03 (0.85 to 1.2) | 13.16  (-2.95 to 20.71) | 11.75  (0.78 to 18.22) | 0.99 (0.82 to 1.16) |
| Palau | 665.93  (27.53 to 921.13) | 1817.49  (-739.39 to 3079.14) | 1.57 (1.43 to 1.72) | 13.46  (0.54 to 18.69) | 11.06  (1.19 to 16.68) | 1.63 (1.48 to 1.78) |
| Palestine | 1160.98  (-417.17 to 1940.27) | 352.60  (-56.56 to 507.34) | -1.63 (-1.73 to -1.53) | 23.68  (-8.53 to 39.67) | 18.27  (4.67 to 27.70) | -1.64 (-1.74 to -1.55) |
| Panama | 556.71  (-50.27 to 856.45) | 165.17  (26.57 to 248.92) | -0.31 (-0.61 to -0.01) | 11.14  (-1.02 to 17.23) | 6.99  (-1.13 to 10.06) | -0.31 (-0.62 to 0) |
| Papua New Guinea | 194.65  (48.54 to 268.72) | 768.12  (-269.14 to 1312.19) | 0.5 (0.35 to 0.64) | 3.91  (0.98 to 5.42) | 19.23  (-7.38 to 31.91) | 0.5 (0.36 to 0.64) |
| Paraguay | 676.39  (-236.07 to 1138.04) | 212.24  (48.96 to 328.52) | -0.97 (-1.09 to -0.85) | 13.86  (-4.82 to 23.29) | 5.78  (1.54 to 8.30) | -0.96 (-1.09 to -0.84) |
| Peru | 289.65  (-25.38 to 423.15) | 161.72  (-57.93 to 268.43) | -1.65 (-2.13 to -1.17) | 5.84  (-0.51 to 8.56) | 11.24  (0.80 to 16.89) | -1.71 (-2.21 to -1.22) |
| Philippines | 254.19  (-37.63 to 381.70) | 654.72  (-586.75 to 1140.80) | -0.06 (-0.14 to 0.03) | 4.94  (-0.70 to 7.46) | 7.09  (-2.86 to 11.50) | -0.01 (-0.09 to 0.07) |
| Poland | 678.39  (-500.72 to 1092.70) | 252.78  (-36.87 to 352.29) | -6.04 (-6.34 to -5.74) | 13.31  (-9.65 to 21.43) | 23.47  (-5.36 to 39.66) | -6.07 (-6.36 to -5.79) |
| Portugal | 1348.27  (-51.51 to 1711.28) | 154.75  (-24.13 to 229.87) | -2.4 (-2.86 to -1.93) | 27.80  (-1.05 to 35.30) | 3.52  (0.17 to 5.76) | -2.32 (-2.79 to -1.86) |
| Puerto Rico | 290.51  (-38.95 to 421.17) | 191.11  (18.52 to 294.63) | -2.68 (-3.17 to -2.19) | 5.96  (-0.80 to 8.66) | 9.45  (-0.69 to 15.89) | -2.71 (-3.2 to -2.22) |
| Qatar | 355.83  (33.65 to 512.62) | 184.62  (10.33 to 302.98) | -4.34 (-4.94 to -3.73) | 7.26  (0.68 to 10.50) | 24.05  (-2.77 to 39.20) | -4.47 (-5.11 to -3.83) |
| Republic of Korea | 470.26  (-48.03 to 725.55) | 76.79  (9.52 to 117.75) | -2.7 (-3.02 to -2.38) | 9.19  (-0.94 to 14.29) | 5.70  (-1.42 to 9.46) | -2.63 (-2.97 to -2.3) |
| Republic of Moldova | 146.69  (-56.40 to 241.13) | 771.42  (-176.31 to 1093.12) | -0.19 (-0.65 to 0.27) | 2.90  (-1.10 to 4.85) | 17.31  (-0.81 to 28.25) | -0.16 (-0.65 to 0.34) |
| Romania | 713.39  (-219.30 to 976.45) | 560.39  (-49.24 to 789.08) | -2.32 (-2.75 to -1.89) | 14.60  (-4.48 to 20.01) | 4.92  (-0.33 to 7.86) | -2.29 (-2.74 to -1.84) |
| Russian Federation | 816.68  (32.14 to 1072.64) | 833.20  (-61.27 to 1140.63) | -2.94 (-3.83 to -2.04) | 16.88  (0.79 to 22.18) | 15.67  (-2.57 to 21.94) | -2.91 (-3.83 to -1.98) |
| Rwanda | 1266.34  (105.97 to 1562.17) | 142.41  (-34.45 to 260.86) | -4.12 (-4.81 to -3.43) | 26.18  (2.27 to 32.35) | 9.70  (-3.07 to 16.13) | -4.22 (-4.92 to -3.51) |
| Saint Kitts and Nevis | 292.62  (-2.26 to 505.05) | 186.18  (10.19 to 288.64) | -4.03 (-4.63 to -3.42) | 5.86  (-0.03 to 10.20) | 25.01  (-1.39 to 40.60) | -3.88 (-4.47 to -3.29) |
| Saint Lucia | 483.29  (4.67 to 678.70) | 143.01  (-31.04 to 226.95) | -2.11 (-2.32 to -1.9) | 9.63  (0.13 to 13.58) | 7.81  (-1.94 to 13.10) | -2.09 (-2.31 to -1.88) |
| Saint Vincent and the Grenadines | 263.37  (-55.17 to 382.85) | 271.65  (-72.29 to 425.07) | -1.84 (-2 to -1.68) | 5.36  (-1.11 to 7.83) | 4.48  (-1.38 to 7.82) | -1.69 (-1.86 to -1.52) |
| Samoa | 444.42  (-72.78 to 622.94) | 1070.38  (-220.73 to 1819.91) | 1.78 (1.63 to 1.92) | 8.94  (-1.45 to 12.59) | 8.85  (-1.99 to 16.86) | 1.82 (1.67 to 1.97) |
| San Marino | 669.06  (-132.07 to 1087.44) | 60.92  (3.62 to 108.63) | -1.91 (-2.19 to -1.63) | 13.77  (-2.74 to 22.59) | 18.96  (0.35 to 28.21) | -1.93 (-2.24 to -1.62) |
| Sao Tome and Principe | 140.49  (2.43 to 212.04) | 182.95  (-68.15 to 340.96) | 0.95 (0.66 to 1.25) | 2.87  (0.06 to 4.37) | 7.47  (-3.05 to 14.22) | 1.01 (0.72 to 1.31) |
| Saudi Arabia | 135.86  (-33.20 to 214.51) | 1145.45  (-260.81 to 1933.09) | 2 (1.74 to 2.27) | 2.73  (-0.67 to 4.32) | 4.57  (-0.78 to 7.90) | 1.98 (1.73 to 2.24) |
| Senegal | 701.69  (-198.39 to 1134.60) | 181.95  (-52.35 to 312.64) | -0.69 (-0.83 to -0.55) | 14.25  (-4.01 to 23.15) | 10.34  (-0.61 to 16.83) | -0.73 (-0.87 to -0.59) |
| Serbia | 223.19  (-71.60 to 363.19) | 340.88  (13.81 to 500.03) | -3.15 (-3.53 to -2.78) | 4.55  (-1.44 to 7.45) | 5.29  (-0.14 to 8.42) | -3.16 (-3.56 to -2.75) |
| Seychelles | 711.35  (53.03 to 959.87) | 396.55  (-313.92 to 698.43) | -0.95 (-1.17 to -0.73) | 14.69  (1.19 to 19.85) | 3.91  (-3.70 to 8.44) | -0.84 (-1.05 to -0.63) |
| Sierra Leone | 504.46  (-306.60 to 828.19) | 219.82  (-122.13 to 400.62) | 0.43 (0.23 to 0.64) | 10.07  (-6.08 to 16.52) | 6.82  (-0.30 to 11.54) | 0.39 (0.18 to 0.6) |
| Singapore | 202.46  (-107.87 to 348.18) | 163.39  (-46.43 to 260.33) | -2.69 (-2.89 to -2.48) | 4.13  (-2.19 to 7.11) | 3.38  (-2.79 to 7.37) | -2.64 (-2.86 to -2.42) |
| Slovakia | 344.13  (-124.78 to 536.93) | 376.22  (26.31 to 543.25) | -3.46 (-3.65 to -3.26) | 6.93  (-2.47 to 10.81) | 6.97  (-1.55 to 12.40) | -3.51 (-3.73 to -3.3) |
| Slovenia | 1035.38  (12.08 to 1374.77) | 121.35  (7.27 to 174.20) | -4.69 (-5.02 to -4.36) | 21.65  (0.38 to 28.71) | 4.46  (-0.84 to 7.53) | -4.87 (-5.23 to -4.51) |
| Solomon Islands | 392.12  (-4.46 to 518.76) | 1423.19  (-387.97 to 2351.14) | 0.86 (0.74 to 0.98) | 8.06  (-0.03 to 10.68) | 2.82  (-0.67 to 5.18) | 0.88 (0.75 to 1.02) |
| Somalia | 1158.41  (-229.39 to 1914.11) | 233.05  (-29.66 to 436.80) | -0.77 (-1 to -0.54) | 23.84  (-4.75 to 39.97) | 7.61  (-2.09 to 12.73) | -0.93 (-1.2 to -0.67) |
| South Africa | 248.69  (-17.92 to 445.14) | 293.16  (-11.75 to 404.87) | -1.43 (-1.91 to -0.95) | 5.07  (-0.34 to 9.16) | 11.79  (5.22 to 15.75) | -1.37 (-1.81 to -0.92) |
| South Sudan | 159.54  (-38.27 to 280.71) | 227.19  (-77.98 to 424.18) | 1.14 (0.82 to 1.46) | 3.22  (-0.76 to 5.71) | 3.27  (0.03 to 4.84) | 1.16 (0.81 to 1.51) |
| Spain | 270.30  (-31.41 to 390.92) | 136.82  (-3.88 to 199.58) | -2.24 (-2.34 to -2.14) | 5.48  (-0.63 to 7.97) | 2.73  (-0.85 to 4.50) | -2.15 (-2.26 to -2.05) |
| Sri Lanka | 467.17  (-164.35 to 699.30) | 343.47  (-189.84 to 628.45) | -1.11 (-1.45 to -0.77) | 9.49  (-3.25 to 14.21) | 2.84  (-0.16 to 4.64) | -1.06 (-1.41 to -0.71) |
| Sudan | 1387.61  (-91.75 to 2171.68) | 877.03  (-37.34 to 1444.06) | -1.51 (-1.56 to -1.47) | 28.16  (-1.89 to 44.17) | 8.10  (-6.39 to 14.31) | -1.55 (-1.59 to -1.52) |
| Suriname | 467.06  (-32.99 to 703.34) | 341.56  (85.24 to 537.88) | -1.25 (-1.53 to -0.97) | 9.49  (-0.66 to 14.47) | 4.57  (-2.15 to 7.80) | -1.16 (-1.45 to -0.87) |
| Sweden | 327.90  (-53.34 to 453.40) | 67.81  (-8.02 to 97.74) | -4.63 (-4.82 to -4.44) | 6.88  (-1.14 to 9.54) | 4.66  (-0.44 to 7.99) | -4.79 (-5.01 to -4.57) |
| Switzerland | 330.63  (-113.65 to 469.17) | 74.20  (-17.41 to 106.06) | -4.94 (-5.15 to -4.72) | 6.82  (-2.34 to 9.74) | 3.97  (-2.21 to 7.55) | -4.97 (-5.2 to -4.73) |
| Syrian Arab Republic | 1296.91  (-307.26 to 2014.54) | 1140.82  (-130.49 to 1855.94) | -0.3 (-0.83 to 0.24) | 25.52  (-6.09 to 39.72) | 2.47(-1.22 to 4.68) | -0.11 (-0.69 to 0.46) |
| Taiwan (Province of China) | 110.98  (52.04 to 153.63) | 120.36  (53.39 to 170.83) | 0.8 (0.55 to 1.04) | 2.15  (1.01 to 2.99) | 4.35  (-1.96 to 7.79) | 0.97 (0.72 to 1.22) |
| Tajikistan | 732.58  (-57.24 to 982.17) | 503.90  (-41.66 to 733.78) | -2.24 (-2.65 to -1.83) | 14.70  (-1.06 to 19.73) | 4.68  (-0.58 to 8.87) | -2.16 (-2.55 to -1.76) |
| Thailand | 217.44  (-160.79 to 373.04) | 237.71  (-176.49 to 475.75) | -0.77 (-1.38 to -0.16) | 4.38  (-3.17 to 7.53) | 5.40  (-1.29 to 11.10) | -0.67 (-1.24 to -0.09) |
| Timor-Leste | 437.07  (-66.82 to 676.31) | 450.47  (-102.87 to 783.14) | 0.08 (-0.31 to 0.46) | 8.66  (-1.25 to 13.42) | 5.67  (-1.02 to 9.93) | 0.14 (-0.24 to 0.51) |
| Togo | 203.46  (-54.39 to 325.11) | 254.22  (-81.25 to 439.45) | 0.66 (0.4 to 0.93) | 4.14  (-1.11 to 6.63) | 2.79  (-0.56 to 4.65) | 0.7 (0.43 to 0.96) |
| Tokelau | 847.78  (-275.92 to 1486.68) | 1014.49  (-447.80 to 1805.22) | 0.24 (0.11 to 0.38) | 17.47  (-5.68 to 30.76) | 7.14  (-2.80 to 13.60) | 0.25 (0.11 to 0.4) |
| Tonga | 496.5  8(-186.22 to 809.68) | 586.13  (-226.10 to 1022.99) | 0.93 (0.77 to 1.1) | 10.30  (-3.88 to 16.89) | 5.94  (-0.23 to 8.29) | 0.94 (0.78 to 1.1) |
| Trinidad and Tobago | 610.78  (219.90 to 821.30) | 484.41  (180.41 to 737.60) | -1.44 (-1.84 to -1.04) | 12.57  (4.51 to 16.97) | 2.41  (-1.14 to 4.16) | -1.47 (-1.86 to -1.08) |
| Tunisia | 478.41  (31.21 to 709.13) | 465.15  (-33.77 to 778.15) | -0.56 (-0.72 to -0.4) | 9.55  (0.61 to 14.17) | 6.03  (0.35 to 10.25) | -0.51 (-0.69 to -0.34) |
| Turkmenistan | 952.95  (-91.42 to 1239.90) | 928.09  (-162.16 to 1446.17) | -1.52 (-2.37 to -0.66) | 19.26  (-1.69 to 25.08) | 3.79  (-0.57 to 6.71) | -1.41 (-2.27 to -0.54) |
| Tuvalu | 1517.62  (-402.98 to 2376.14) | 1563.66  (-519.48 to 2576.61) | 0.06 (-0.09 to 0.21) | 31.46  (-8.33 to 49.41) | 3.56  (-2.20 to 6.78) | 0.03 (-0.13 to 0.2) |
| Türkiye | 573.43  (-147.05 to 872.44) | 279.33  (-69.52 to 462.65) | -2.66 (-2.87 to -2.46) | 11.63  (-2.99 to 17.83) | 3.08  (-2.54 to 6.04) | -2.64 (-2.85 to -2.43) |
| Uganda | 119.33  (-37.85 to 210.93) | 125.31  (-62.16 to 235.36) | -0.79 (-1.23 to -0.34) | 2.40  (-0.74 to 4.30) | 4.84  (-3.52 to 9.03) | -0.84 (-1.28 to -0.4) |
| Ukraine | 812.84  (-93.67 to 1158.96) | 1010.11  (-134.79 to 1634.46) | -0.68 (-1.38 to 0.02) | 17.01  (-1.93 to 24.25) | 4.12  (-0.00 to 6.62) | -0.7 (-1.42 to 0.03) |
| United Arab Emirates | 467.79  (20.28 to 752.00) | 249.59  (-16.55 to 393.98) | -2.5 (-2.88 to -2.12) | 9.44  (0.42 to 15.26) | 4.88  (-2.03 to 8.41) | -2.66 (-3.06 to -2.25) |
| United Kingdom | 517.67  (13.58 to 678.00) | 178.19  (7.19 to 234.81) | -3.44 (-3.52 to -3.35) | 10.92  (0.24 to 14.33) | 3.50  (-2.34 to 6.36) | -3.49 (-3.57 to -3.4) |
| United Republic of Tanzania | 144.20  (-75.72 to 252.24) | 200.09  (-112.06 to 378.65) | 0.8 (0.6 to 1.01) | 2.86  (-1.49 to 5.02) | 3.74  (-1.39 to 6.94) | 0.79 (0.59 to 0.99) |
| United States of America | 451.63  (112.37 to 594.09) | 273.45  (111.97 to 354.96) | -1.77 (-2.09 to -1.44) | 9.43  (2.35 to 12.44) | 1.80  (-0.47 to 3.33) | -1.77 (-2.11 to -1.43) |
| United States Virgin Islands | 594.48  (-3.19 to 920.37) | 371.80  (67.90 to 611.82) | -1.31 (-1.58 to -1.04) | 12.35  (-0.08 to 19.13) | 2.55  (-0.91 to 4.41) | -1.31 (-1.58 to -1.04) |
| Uruguay | 483.91  (-16.76 to 624.48) | 232.92  (48.87 to 305.71) | -2.44 (-2.79 to -2.08) | 10.12  (-0.33 to 13.10) | 7.59  (-2.78 to 13.16) | -2.46 (-2.82 to -2.1) |
| Uzbekistan | 806.61  (-109.38 to 1053.64) | 867.14  (-148.20 to 1222.21) | -0.05 (-0.45 to 0.35) | 16.10  (-2.11 to 20.99) | 3.11  (-1.77 to 5.31) | 0.01 (-0.41 to 0.44) |
| Vanuatu | 1489.83  (-669.22 to 2498.94) | 1745.77  (-748.60 to 2925.38) | 0.38 (0.33 to 0.43) | 30.53  (-13.76 to 51.53) | 3.93  (0.58 to 6.52) | 0.39 (0.34 to 0.44) |
| Venezuela (Bolivarian Republic of) | 501.72  (90.02 to 678.81) | 639.32  (92.34 to 994.27) | -0.04 (-0.47 to 0.39) | 10.05  (1.82 to 13.63) | 5.41  (-1.68 to 9.16) | -0.01 (-0.44 to 0.42) |
| Viet Nam | 161.93  (-85.69 to 269.60) | 193.41  (-146.20 to 365.88) | 1.06 (0.82 to 1.29) | 3.1  9(-1.64 to 5.32) | 3.69  (-1.06 to 6.38) | 1.23 (0.99 to 1.46) |
| Yemen | 1131.05  (-28.17 to 1804.89) | 843.38  (-38.64 to 1375.29) | -1.26 (-1.47 to -1.05) | 23.40  (-0.59 to 37.39) | 4.44  (-2.45 to 8.11) | -1.33 (-1.56 to -1.1) |
| Zambia | 147.42  (-74.38 to 240.56) | 215.40  (-98.54 to 385.57) | 1.07 (0.89 to 1.24) | 2.95  (-1.46 to 4.85) | 21.16  (-9.41 to 36.49) | 1.09 (0.91 to 1.26) |
| Zimbabwe | 89.90  (-6.88 to 135.44) | 293.64  (17.17 to 495.72) | 4.67 (3.72 to 5.63) | 1.80  (-0.13 to 2.73) | 5.22  (-1.67 to 9.07) | 4.73 (3.76 to 5.7) |

**Table S4** Data on IHD related to Mortality Rate and DALYs Rate attributed to dietary risks over the next 10 years.

| Year | Mortality Rate | DALYs Rate |
| --- | --- | --- |
| 2022 | 9.49  (9.25 to 9.73) | 467.13  (455.92 to 478.34) |
| 2023 | 9.44  (8.95 to 9.93) | 467.13  (445.91 to 488.35) |
| 2024 | 9.37  (8.67 to 10.07) | 467.13  (439.3 to 494.96) |
| 2025 | 9.31  (8.44 to 10.18) | 467.13  (433.98 to 500.28) |
| 2026 | 9.26  (8.23 to 10.29) | 467.13  (429.4 to 504.86) |
| 2027 | 9.21  (8.01 to 10.42) | 467.13  (425.32 to 508.94) |
| 2028 | 9.17  (7.78 to 10.56) | 467.13  (421.61 to 512.65) |
| 2029 | 9.12  (7.54 to 10.7) | 467.13  (418.17 to 516.09) |
| 2030 | 9.07  (7.29 to 10.85) | 467.13  (414.96 to 519.29) |
| 2031 | 9.02  (7.03 to 11.01) | 467.13  (411.94 to 522.32) |
